# Development of Novel Potential Pleiotropic Compounds of Interest in Alzheimer’s Disease Treatment through Rigidification Strategy

**DOI:** 10.3390/molecules26092536

**Published:** 2021-04-26

**Authors:** Cédric Lecoutey, Rémi Legay, Audrey Davis, Jana Sopková-de Oliveira Santos, Patrick Dallemagne, Christophe Rochais

**Affiliations:** Normandie Univ, Unicaen, Cermn, 14000 Caen, France; cedric.lecoutey@unicaen.fr (C.L.); remi.legay@unicaen.fr (R.L.); audrey.davis@unicaen.fr (A.D.); jana.sopkova@unicaen.fr (J.S.-d.O.S.)

**Keywords:** Alzheimer’s disease, acetylcholinesterase, 5-HT_6_R, donecopride, MTDL

## Abstract

The development of Multi-Target Directed Ligand is of clear interest for the treatment of multifactorial pathology such as Alzheimer’s disease (AD). In this context, acetylcholinesterase (AChE) inhibitors have been modulated in order to generate novel pleiotropic compounds targeting a second protein of therapeutic interest in AD. Among them, donecopride was the first example of a dual acetylcholinesterase inhibitor and 5-HT_4_ receptor agonist. In order to explore the structural diversity around this preclinical candidate we have explored the preparation of novel constrained analogs through late-stage rigidification strategy. A series of phenylpyrazoles was prepared in a late-stage functionalization process and all compounds were evaluated in vitro towards AChE and 5-HTRs. A docking study was performed in order to better explain the observed SAR towards AChE, 5-HT_4_R and 5-HT_6_R and this study led to the description of novel ligand targeting both AChE and 5-HT_6_R.

## 1. Introduction

**Figure 1:**
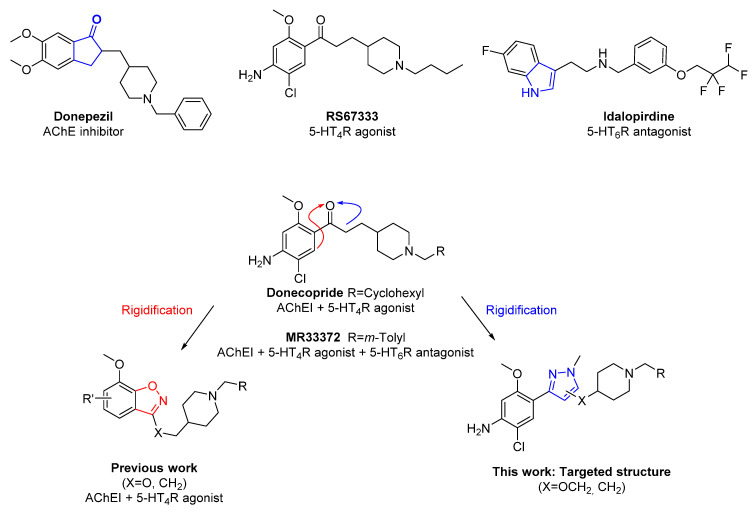
Structures of reference ligands and MTDL (Donecopride, MR33372, benzisoxazole) and targeted structure.

In the field of neurodegenerative diseases, the prototype of multifactorial pathologies, the development of Multi-Target Directed Ligands (MTDLs) offered great promise and therapeutic opportunities [1]. Indeed, these compounds are rationally designed to display multiple activities by modulating several biological targets of interest in the treatment of a specific disease. Chosen to demonstrate a therapeutic synergy, the association of these biological targets needs to be rigorously determined [2]. In the field of Alzheimer’s disease (AD), multiple in vitro or in vivo MTDL candidates have been generated over the last years and could lead to preclinical or clinical opportunities [3]. Because of its validated interest in the treatment of AD symptoms, acetylcholinesterase (AChE) inhibitors, such as donepezil, have been chosen as a starting point to develop novel analogs with a second biological target such as receptors [4]. As an example, we recently described the preclinical candidate donecopride, the first MTDL able to demonstrate AChE inhibition and activating serotoninergic receptors 5-HT_4_ (Figure 1) [5,6,7].

This secondary target was chosen because of the ability of 5-HT_4_R agonists to favor the “non-amyloidogenic” cleavage of the amyloid precursor protein (APP) by α-secretase, inducing then a decrease in amyloid pathology. [8] This positive effect has already been demonstrated with the use of RS67333 a reference 5-HT_4_R agonist, in primary neurons, [9] and led to the in vivo improvement of memory in several animal models of AD [10]. Indeed, in the context of AD, the activation of 5-HT_4_Rs with agonist has led to the increase in neurotransmitter release as well as a clear impact of amyloid load in transgenic mice models after chronic administration. Several 5-HT_4_R agonists have been studied in clinical trials in recent years to demonstrate both symptomatic and disease-modifying effect against AD [11]. Beside this receptor subtypes, other serotonin receptors have been studied in the field of neurodegenerative diseases [12], such as the 5-HT_6_R [13,14]. Specifically, 5-HT_6_R antagonists increase extracellular levels of acetylcholine, glutamate, and norepinephrine in forebrain regions, whereas activation of 5-HT_6_R inhibits corticostriatal glutamatergic transmission. In the end, these various elements (notably, distribution in limbic areas, modulation of different systems of neurotransmission) are in favor of the implication of 5-HT_6_R in the modulation of the cognitive processes as well as mood regulation and of numerous behaviors (eating behavior, addictive behavior, etc.). Indeed, they appear as a valuable target to treat cognitive impairments since their blockade confers to 5-HT_6_R antagonists’, such as idalopirdine (Figure 1), procognitive effects. [15] It is important to note that during the clinical trials the potential synergistic effect to act simultaneously on 5-HT_6_R and AChE has been evaluated since idalopirdine has been tested along with donepezil [16].

In this context, we recently described initial modulations of donecopride by introducing a series of substituent on the piperidine ring, which yielded the description of a first 5-HT_4_R agonist and 5-HT_6_R antagonist, [17] and MR33372, the first compound able to modulate simultaneously AChE, 5-HT_4_R and 5-HT_6_R [18]. In parallel with these first works, we also decided to assess the impact of conformational restriction on the aromatic ring, inspired by both bicyclic donepezil and idalopirdine (Figure 1). Indeed, such restriction are classical in drug discovery [19], and we recently described the preparation and biological evaluation of benzisoxazole analog possessing an oxygen or a methylene linker [20]. We would like to describe in this article, a novel rigidification strategy with the construction of a novel heterocycle between the ketone and its alpha-position. The preparation of novel phenylpyrazole derivatives possessing either a methylene or an oxygen linker (Figure 1), as well as their biological evaluation against AChE and both 5-HT_4_R and 5-HT_6_R will be described and compared with other members of this series.

## 2. Results

### 2.1. Chemistry

**Scheme 1:**
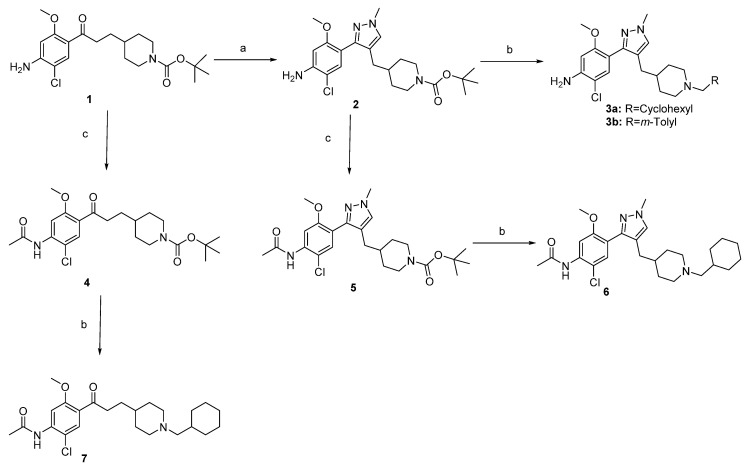
Synthetic pathways for access to compounds 2–7. Reagents and conditions: (a) i. DMF.DMA, Rflx, ii. CH_3_NHNH_2_, EtOH, Rflx, mw, 38%; (b) i. TFA, DCM, TA, ii. RCH_2_Br, K_2_CO_3_, DMF, or acetone, 110 °C, 31–46%; (c) Ac_2_O, rt, 24 h, 78–83%.

The access to targeted compounds was achieved starting from Boc-protected benzophenone **1** that we previously described (Scheme 1) [5]. The latter was treated by DMF–DMA [21] to generate an intermediate dimethyl enamines, which was not isolated, but reacted in boiling ethanol with methylhydrazine to generate the expected pyrazole **2** with 38% yield [22,23]. In order to obtain the final derivatives, the Boc protecting group was removed with TFA, and the resulted piperidine was alkylated with the appropriate bromide to generate the final cyclohexyl and *m*-tolyl substituted analog **3a**–**b**. These particular substituents were chosen for a better comparison with either donecopride and MR33372. In order to verify the impact of the aniline both on the reactivity and the biological activities, the corresponding acetamides **4**–**5** were obtained in acetic anhydride with good yields. Following the same procedure, the cyclohexyl substituted analogs **6** and **7** were prepared after deprotection and alkylation (Scheme 1).

**Scheme 2:**
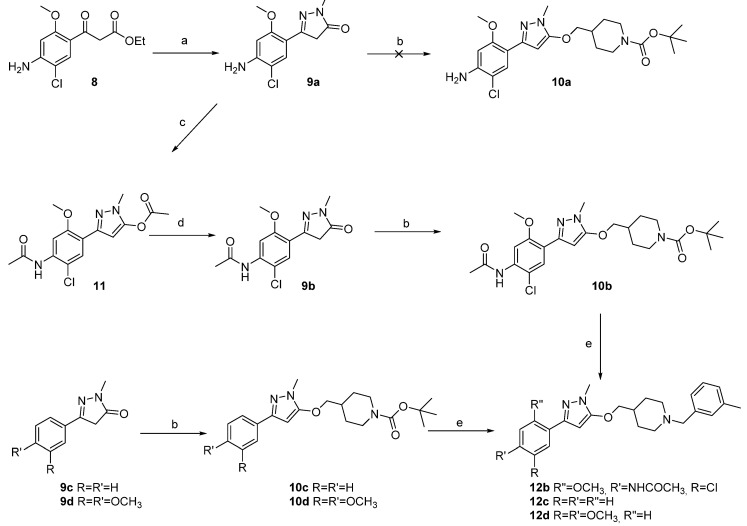
Synthesis pathways for access to compounds **9**–**12**. Reagents and conditions: (a) CH_3_NHNH_2_, EtOH, 160 °C, mw, 57%; (b) *N*-Boc 4-iodomethylpiperidine, K_2_CO_3_, DMF, 60 °C, 21–27%; (c) Ac_2_O, 94%, (d) LiOH, EtOH/H_2_O, Rfx, 76%, (e) i. TFA, DCM, TA, ii. *m*-TolylCH_2_Br, K_2_CO_3_, acetone, 110 °C, 32–67%.

As already described in the benzisoxazole [20] and donecopride series [6], the influence of the linker could be of great importance on the ability of the ligand to bind to AChE. We then investigated the possibility to modulate both the linker and its position on the pyrazole ring. Indeed, the β-ketoester **8** is a crucial intermediate in the preparation of the benzophenone **1** and was then already available [6]. Afterwards, **8** was treated with methylhydrazine in refluxing ethanol to obtain the pyrazolone **9a** with 57% yield (Scheme 2). Unfortunately, the O-alkylation procedure tested was ineffective to generate the expected piperidine **10a**, due to the reactivity of the aniline moiety. The pyrazolone **9a** was then treated with acetic anhydride to obtain the ester **11** with 94% yield, before being hydrolyzed with LiOH to generate the acetamide **9b**. The latter was then alkylated with *N*-Boc 4-iodomethylpiperidine in DMF to generate this time the expected ether **10b** with good conversion. Finally, the protecting group was removed with trifluoroacetic acid, and the *m*-tolyl analog **12b** was obtained after an *N*-alkylation reaction with the corresponding bromide. Unfortunately, all attempts aiming at deprotecting the *N*-acetamide group of **12b** failed. In order to verify this time the impact of the substitution of the aromatic ring, commercial pyrazolone **9c**–**d** were engaged in the same procedure. The 3-4-dimethoxyphenyl group of **9d** was chosen by analogy with donepezil, a reference AChE inhibitor (AChEI). The expected *m*-tolyl analogs **12c**–**d** were obtained in a two-step synthesis.

### 2.2. In Vitro Results

The inhibitory activity of novel analogs towards *h*AChE was evaluated according to the Ellman test [24]. Their affinity for human 5-HT_4_R and 5-HT_6_R was assessed using a radioligand displacement assay (Table 1). In these tests, donepezil (DPZ) was used as a reference AChEI, RS67333 as a 5-HT_4_R ligand, and idalopirdine as a 5-HT_6_R ligand, respectively.

As already introduced, we demonstrated that pharmacomodulation of the preclinical candidate donecopride could greatly affect the ability of its analog to interact with ChE and 5-HT receptors. Indeed, in our initial study [6], we showed that its ester or amide analogs lose their ability to inhibit AChE. The same tendency has also been observed in the benzisoxazole family with a clear impact of the modulation of the benzenic position on both AChE and 5-HT_4_R binding. [20] In this context, we decided to explore the modulation of the α-position of the ketone and the possibility to generate conformationally constrained derivatives to increase the elucidation of SAR around donecopride. The preparation of first series of phenylpyrazoles **3a**–**b** and **6**, was then achieved from the advance intermediate **1**. In this synthesis, the protection of the basic nitrogen with a Boc group is mandatory to obtain the pyrazole cycle, since all attempts to modulate the final donecopride analog in a late stage process failed. For this reason, we also explored in parallel the impact of the protection of the aniline moiety with an acetamide group, with no clear benefit in terms of synthesis of compound **6**. The preparation of the Boc protected **2** was, however, of interest since it allowed us to introduce in a convergent way both the cyclohexyl and the *m*-tolyl moieties. Indeed if the cyclohexyl present in donecopride yielded low nanomolar affinities for both AChE and 5-HT_4_R, [6] its replacement by a *m*-tolyl generated triple activity this time towards 5-HT_6_R [18]. If MR33372 possesses affinities in the hundred nM range for the three targets, this compound demonstrated an in vivo anti-amnesic effect in a model of scopolamine induced deficit.

Compared with donecopride, the introduction of the acetamide group on **7** had little impact on AChE inhibition (IC_50_ = 16 vs. 34 nM respectively), but greatly affected the affinity for 5-HT_4_R (Table 1). The introduction of the methylpyrazole ring on **3a** led to a complete loss of activities for all targets. Interestingly, however, the introduction of the *m*-tolyl moiety is again of interest for **3b**. Compared to MR33372 this time, **3b** is a slightly better AChEI (IC_50_ = 161 vs. 111 nM respectively) and possesses a decreased affinity for 5-HT_6_R. Contrary, the conformational restriction greatly affects the ability of the compound to bind to 5-HT_4_R. Due to its synthetic accessibility from intermediate **8**, a second series of ether substituted analog **12b**–**d** was generated bearing the *m*-tolyl substituent previously identified. Unfortunately, this modulation led to a complete loss of activities for the three targets.

### 2.3. In Silico Results

**Figure 2:**
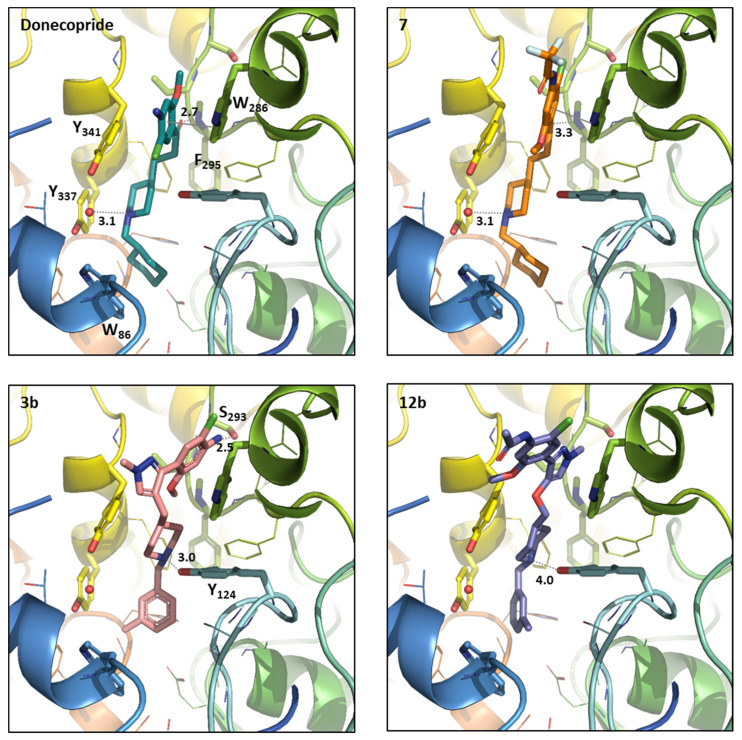
Donecopride (green) position compared to the compound **7** (orange), **3b** (pink), and **12b** (purple) positioned in *h*AChE binding sites using the docking studies. The compound and the selected side chains of the binding site residues are in stick and the protein in ribbon representation. This figure was made with PYMOL (DeLano Scientific, South San Francisco, CA, USA, 2002, San Carlo, Redwood City, CA, USA) and the protein is presented in the same orientation in all four images.

In order to better understand the impact of these SAR on AChE binding, a docking study was performed with **3b** in comparison with donecopride (Figure 2). The docking study was carried out with the aim to predict the AChE inhibition of synthesized compounds compared to donecopride, the initial scaffold of the carried out modulations. The docking study into the *h*AChE active site (PDB code: 4EY7) [25] was performed using Gold 5.7.2 software and the ligand 3D models were built from donecopride X-ray structure solved previously. Firstly, the correctness of employed docking procedure was checked by redocking the co-crystallized ligand, donepezil. Gold program regained the crystallographic donepezil position with a RMSD smaller to 1 Å and with a score fit value of 110.02 [6]. Next, the docking procedure was applied to donecopride and to the newly synthesized compounds (**3b**, **7,** and **12b**). The docking generated positions closed to donepezil one for donecopride (score fit ~105.14, Figure 2) and for compound **7** (score fit ~ 117.63, Figure 2) and **12b** (score fit~86.79, Figure 2). Indeed, donecopride and compound **7** under the docking results reproduces well the donepezil key binding interactions: (i) the charged nitrogen of the piperidine ring is oriented in a position suitable for an Hbond with the water molecule in the proximity of Tyr_337_ and Tyr_341_, (ii) the carbonyl group of both ligands forms an Hbond with NH of the Phe_295_ backbone, (iii) the benzene ring is positioned in a parallel way to the Trp_286_ indole ring to favor the π-stacking interaction and (iv) the cyclohexane ring occupied the donepezil benzyl ring place in neighboring of Trp_86_. Even if compound **12b** was positioned systematically with the substituted phenyl ring towards the binding cavity’s bottom, its position was higher comparing to previous three compounds. Consequently, **12b** lost, more to the loss of Hbond due to carbonyl group absence in its scaffold, also the interaction through the piperidine’s protonated nitrogen as well as π-stacking with Trp_86_ (Figure 2).

However, the first docking does not produce conclusive results for compound **3b**. Compound **3b** was systematically placed with aniline moiety at the bottom of the binding site cavity. Therefore, a second docking with a greater number of docking cycles was tested for compound **3b**. This docking generated even some more logical poses of compound **3b** with aniline moiety placed at the binding cavity entrance. The generated solution with good orientation of aniline moiety corresponded to the score fit 91.78 is present in Figure 2.

The compound **3b** docked in the *h*AChE active site higher compared to the donecopride in the same way as compound **12b**. Like **12b**, **3b** compound lost the interaction through the piperidine’s protonated nitrogen, π-stacking with Trp_86,_ and the Hbond interaction due to carbonyl group absence in its scaffold (Figure 2). However, on the other hand with compound **12b**, **3b** establishes new interactions through hydrogen bonds with the hydroxy group of Tyr_124_ and Ser_293_.

Based on this study we could state that the introduction of the methylpyrazole ring led to the loss of one of the crucial hydrogen bonds with the backbone NH of Phe_295_. In this series, the classical cation-π interaction with Tyr_337_ is not enough to maintain the affinity of the cyclohexyl **3a**. The replacement of the cyclohexyl by the aromatic *m*-tolyl led to clear impact on AChE inhibition surely by increasing the stacking of the aromatic ring of **3b** with Phe_86_ (Figure 2). Both interactions as well as hydrophobic interactions in the aromatic gorge could then justify the interesting properties of the novel phenyl pyrazole **3b**.

**Figure 3:**
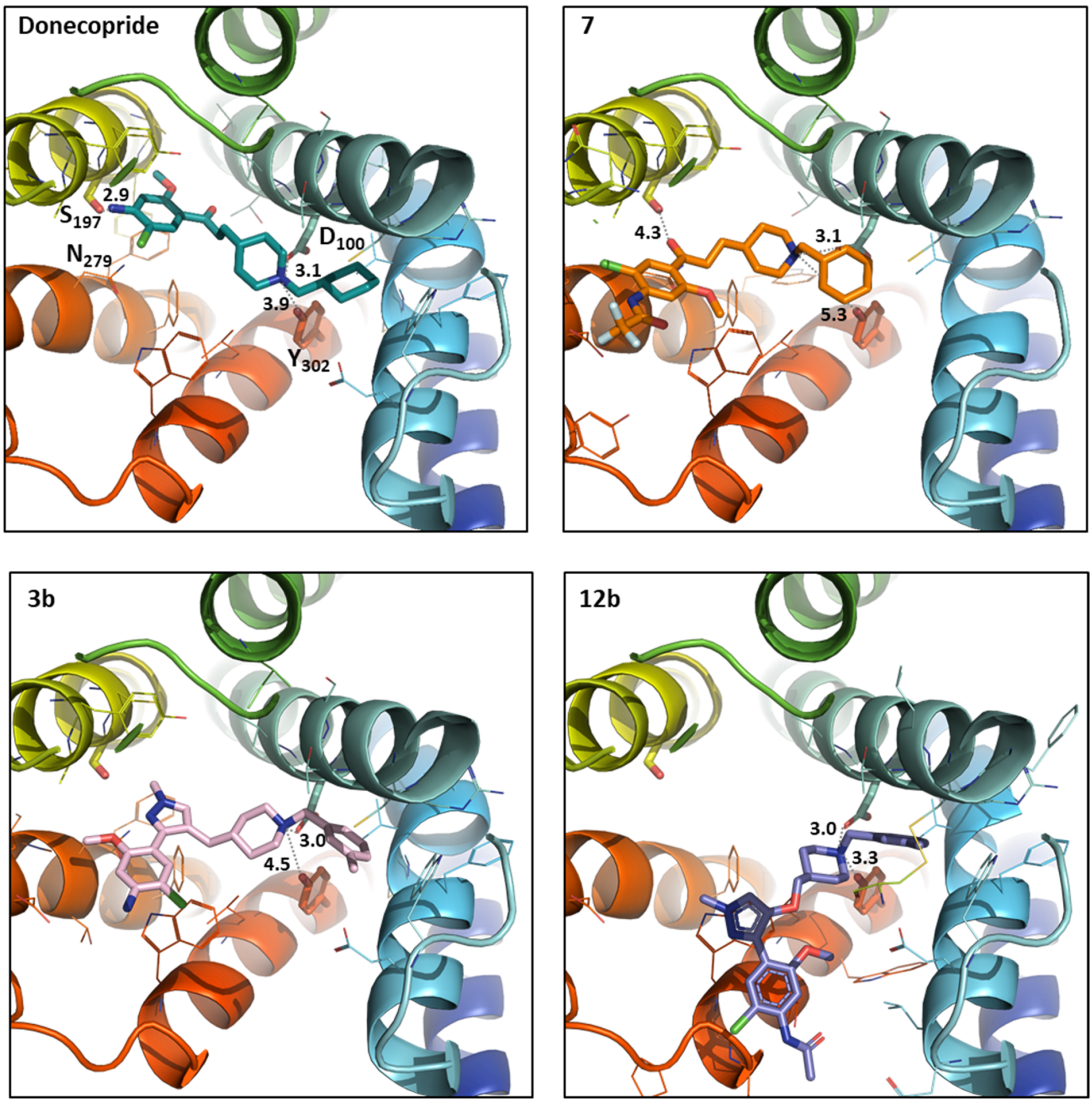
Donecopride (green) position compared to the compound **7** (orange), **3b** (pink), and **12b** (purple) positioned in *h*5-HT_4_R binding sites using the docking studies. The compound and the selected side chains of the binding site residues are in stick and the protein in ribbon representation. This figure was made with PYMOL (DeLano Scientific, 2002, San Carlo, CA, USA) and the protein is presented in the same orientation in all four images.

In order to better understand their worst profile on serotonergic receptors, docking studies were performed in a homology model of the 5-HT_4_ and 5-HT_6_ receptors. The docking results on 5-HT_4_R for compounds **7**, **3b,** and **12b** were compared to donecopride one [6]. From donecopride docking, the selected pose was that where donecopride is placed horizontally in the cavity interacting on one side through an H-bond with Asp_100_ on the transmembrane helix 3 (TM3, consistent with the constraint used during docking) and on other side through another H-bond with Ser_197_ on TM5 (in yellow in Figure 3). The donecopride’s group assuring this second H-bond is NH_2_ substituent on the benzene ring. Therefore, the addition of the substituent on NH_2_ group as well as the lengthening of the connector between the benzene cycle and the piperidine increase the compounds’ length and the compounds can no longer be placed horizontally as donecopride. They take a curve position (Figure 3) and lost the interaction with TM5 in binding site. For compound **7**, the position of its carbonyl group allowed it to maintain some electrostatic interactions with Ser_197_ and Asn_279_, which is impossible for compound **12b** and **3b** through the replacement of this carbonyl group by the methylpyrazole ring. Hence, the hypothesis of their loss of affinity.

**Figure 4:**
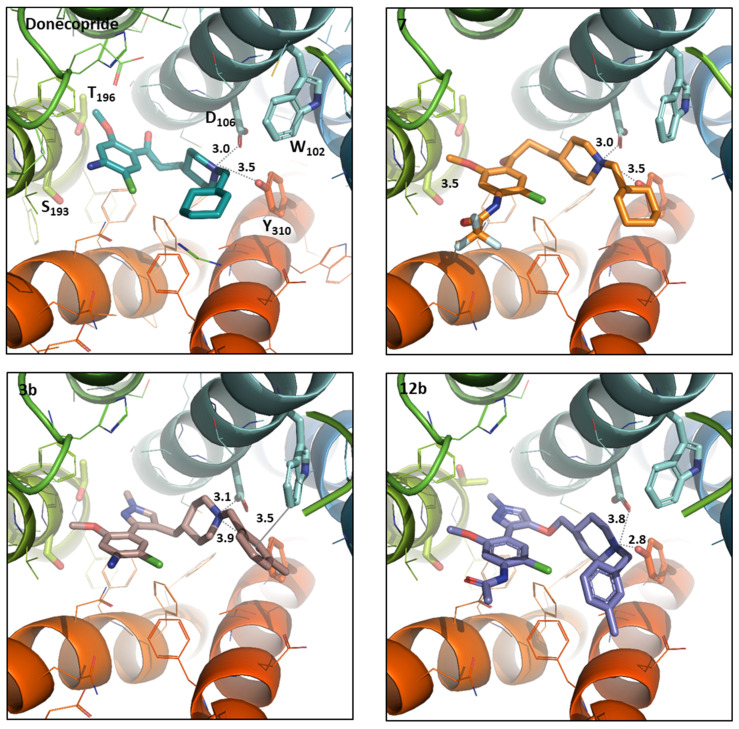
Donecopride (green) position compared to the compound **7** (orange), **3b** (pink) and **12b** (purple) positioned in *h-*5HT_6_R binding sites using the docking studies. The compound and the selected side chains of the binding site residues are in stick and the protein in ribbon representation. This figure was made with PYMOL (DeLano Scientific, 2002, San Carlo, CA, USA) and the protein is presented in the same orientation in all four images.

The comparison of the binding site of 5-HT_6_R and 5-HT_4_R showed that the various amino acids change and so modify the binding site topology. For example, in the 5-HT_6_R, two tryptophan residues are in proximity of TM3 helix, Trp_102_ on TM3, and Trp_92_ in the ECL1 loop (Figure 4), and these two residues with a bulky lateral chains obstruct the space between the helices TM2 and TM3, which leads to the ligands no longer accessing this pocket, unlike the 5-HT_4_R. The binding cavity in 5-HT_6_R is smaller than that of 5-HT_4_R and therefore the ligands must take curved conformations to be able to access Asp_106_. A supplementary interaction through π stacking with Trp_102_ was only observed for ligand **3b** (Figure 4), which could explain the micromolar binding affinity of this ligand to 5-HT_6_R.

## 3. Experimental

### 3.1. Chemistry

#### 3.1.1. General Methods

All chemical reagents and solvents were purchased from commercial sources and used without further purification. Melting points were determined on a STUART SMP50 melting point apparatus (Cole-Parmer, Vernon Hills, IL, USA). ^1^H, ^13^C, and ^19^F NMR spectra were recorded on a BRUKER AVANCE III 400 MHz (Bruker, Billerica, MA, USA) apparatus with chemical shifts expressed in parts per million downfield from TMS as an internal standard and coupling in Hertz. IR spectra were recorded on a Perkin-Elmer BX FT-IR apparatus (Perkin Elmer, Wellesley, MA, USA) using KBr pellets. High-resolution mass spectra (HRMS) were obtained by electrospray on a BrukermaXis (Bruker, Billerica, MA, USA). The purities of all tested compounds were analyzed by LC−MS, with the purity all being higher than 95%. Analyses were performed using a Waters Alliance 2695 (Waters corporation, Milford, MA, USA) as separating module (column XBridge C18 2.5 μM/4.6 × 50 mM) using the following gradients: A (95%)/B (5%) to A (5%)/B (95%) in 4.00 min. This ratio was hold during 1.50 min before return to initial conditions in 0.50 min. Initial conditions were then maintained for 2.00 min (A = H_2_O, B = CH_3_CN; each containing HCOOH: 0.1%). MS were obtained on a SQ detector by positive ESI.

#### 3.1.2. Synthesis of Compounds (**2**–**7**, **9**–**12**)

*tert-butyl 4-((3-(4-amino-5-chloro-2-methoxyphenyl)-1-methyl-1H-pyrazol-4-yl)methyl)piperidine-1-carboxylate***2**. An amount of 375 mg of compound **1** (0.947 mmol) were refluxed in 5 mL of DMF.DMA overnight. After evaporation under reduced pressure, the residue was dissolved in 10 mL of EtOH and 26 mL of methylhydrazine (3.788 mmol, 4 eq) were added. The mixture was refluxed for 4 h. After concentration, the new residue was dissolved in 20 mL of EtOH and 645 mg of ZnCl_2_ (4.735 mmol, 5 eq) were added. The new reactional mixture was refluxed for 2 h and then concentrated. The residue was dissolved in DCM and washed 3 times with water. The organic phase was dried on MgSO_4_, concentrated and purified on neutral Al_2_O_3_ to afford 155 mg of product **2** as a brown solid. Yield = 38%; m.p. = 67–69 °C; ^1^H-NMR (CDCl_3_, 400 MHz): 7.17 (s, 1H), 7.15 (s, 1H), 6.34 (s, 1H), 4.10 (br s, 2H), 3.99 (br s, 2H), 3.87 (s, 3H), 3.71 (s, 3H), 2.57 (t, *J* = 13.0 Hz, 2H), 2.29 (d, *J* = 7.0 Hz, 2H), 1.54 (d, *J* = 13.1 Hz, 2H), 1.45−1.38 (m, 10H), 0.97 (qd, *J* = 12.2 Hz, *J’* = 4.2 Hz, 2H); ^13^C-NMR (CDCl_3_, 100 MHz): 156.8, 154.9, 147.4, 143.5, 131.7, 129.3, 118.7, 114.4, 110.7, 98.7, 79.2, 55.6, 44.0 (2C), 38.9, 37.0, 32.0 (2C), 31.1, 28.5 (3C); MS [M + H]^+^ = 434.86; IR (KBr, cm^−1^): 3468, 3351, 2931, 1686, 1624, 1461, 1444, 1426, 1365, 1247, 1211, 1173, 1211.

##### General Procedure for Acetylation

The aniline was stirred in anhydric acetic (6 mL·mmol^−1^) at room temperature overnight. The mixture was concentrated under reduced pressure. The residue i was then dissolved in EtOAc and washed twice with aq. NaHCO_3_ saturated and once with water. The organic phase was dried, filtrated, and evaporated in vacuo. The crude product is purified on silica gel.

*tert-butyl 4-(3-(4-acetamido-5-chloro-2-methoxyphenyl)-3-oxopropyl)piperidine-1-carboxylate***4**. From 150 mg of compound **1** (0.379 mmol), 129 mg of compound **4** were obtained as a white solid (gradient of elution: DCM to DCM/EtOAc 9/1). Yield = 78%; m.p. = 123–125 °C; ^1^H-NMR (CDCl_3_, 400 MHz): 8.26 (s, 1H), 7.80 (br s, 1H), 7.77 (s, 1H), 4.07 (br s, 2H), 3.90 (s, 3H), 2.94 (m, 2H), 2.65 (m, 2H), 2.26 (s, 3H), 1.66–1.56 (m, 4H), 1.43 (s, 9H), 1.42 (m, 1H), 1.08 (m, 2H); ^13^C-NMR (CDCl_3_, 100 MHz): 199.6, 168.7, 158.5, 154.9, 138.8, 130.6, 123.4, 113.7, 104.0, 79.2, 56.0, 44.0 (2C), 40.9, 35.7, 32.1 (2C), 30.9, 28.5 (3C), 25.2; MS [M + H]^+^ = 438.84; IR (KBr, cm^−1^): 3323, 2975, 2930, 2853, 1690, 1674, 1599, 1578, 1513, 1451, 1402, 1242, 1163, 1017. HRMS [M + H + Na]^+^calcd for C_22_H_31_ClN_2_NaO_5_, 461.1819, exp 461.1813.
*tert-butyl 4-((3-(4-acetamido-5-chloro-2-methoxyphenyl)-1-methyl-1H-pyrazol-4-yl)methyl)piperidine-1-carboxylate***5**. From 38 mg of compound **2** (0.088 mmol), 35 mg of compound **5** were obtained as a white solid (gradient of elution: DCM to DCM/EtOAc 6/4). Yield = 83%; m.p. = 147–149 °C; ^1^H-NMR (CDCl_3_, 400 MHz): 8.20 (s, 1H), 7.71 (s, 1H), 7.32 (s, 1H), 7.17 (s, 1H), 3.98 (br s, 2H), 3.88 (s, 3H), 3.81 (s, 3H), 2.56 (t, *J* = 12.8 Hz, 2H), 2.31 (d, *J* = 7.0 Hz, 2H), 2.26 (s, 3H), 1.53 (d, *J* = 13.2 Hz, 2H), 1.45−1.38 (m, 10H), 0.96 (qd, *J* = 12.2 Hz, *J’* = 4.3 Hz, 2H); ^13^C-NMR (CDCl_3_, 100 MHz): 168.4, 155.7, 154.8, 154.4, 144.7, 134.4, 127.4, 119.3, 113.8, 104.4, 86.0, 79.5, 75.7, 55.9, 43.5 (2C), 36.1, 33.7, 28.6 (2C), 28.5 (3C), 25.1; MS [M + H]^+^ = 476.82; IR (KBr, cm^−1^): 3420, 2930, 1688, 1584, 1527, 1450, 1388, 1244, 1161.
*5-(4-amino-5-chloro-2-methoxyphenyl)-2-methyl-2,4-dihydro-3H-pyrazol-3-one***9a**. A total of 300 mg of compound **8** (1.107 mmol), 54 µL of methylhydrazine (0.996 mmol, 0.9 eq), and 3 mL of EtOH were stirred under microwaves irradiation at 160 °C for 45 min. The solvent was eliminated under reduced pressure and the residue was purified on silica gel (gradient of elution: DCM to EtOAc) to afford 143 mg of compound 9a as a white solid. Yield = 57%; m.p. = 203–205°C; ^13^C-NMR (CDCl_3_, 400 MHz): 7.81 (s, 1H), 6.27 (s, 1H), 4.31 (br s, 2H), 3.78 (s, 3H), 3.68 (s, 2H), 3.36 (s, 3H); ^13^C-NMR (CDCl_3_, 100 MHz): 172.5, 157.5, 152.4, 145.6, 128.0, 111.7, 111.4, 98.0, 55.6, 42.1, 31.3; MS [M + H]^+^ = 253.92; IR (KBr, cm^−1^): 3448, 3331, 2958, 2229, 1677, 1631, 1603, 1572, 1458, 1348, 1214, 1049; HRMS [M + H]^+^calcd for C_11_H_13_ClN_3_O_2_, 254.0696, exp 254.0693.
*3-(4-acetamido-5-chloro-2-methoxyphenyl)-1-methyl-1H-pyrazol-5-yl acetate***11.** A total of 200 mg of compound **9a** (0.80 mmol) were stirred in 2 mL of anhydric acetic at room temperature overnight. Water was added to obtain a white suspension. This latter was filtrated and washed with water. A total of 250 mg of product 11 were then obtained as a white solid. Yield = 94%. m.p. = 191–193°C; ^1^H-NMR (CDCl_3_, 400 MHz): 8.21 (s, 1H), 7.92 (s, 1H), 7.68 (s, 1H), 6.61 (s, 1H), 3.89 (s, 3H), 3.73 (s, 3H), 2.35 (s, 3H), 2.25 (s, 3H); ^13^C-NMR (CDCl_3_, 100 MHz): 168.4, 166.1, 155.8, 145.1, 144.8, 134.6, 127.3, 118.8, 113.6, 104.3, 95.5, 55.9, 34.7, 25.1, 20.7; MS [M + H]^+^ = 337.92; IR (KBr, cm^−1^): 3422, 2945, 1787, 1772, 1699, 1585, 1511, 1483, 1450, 1352, 1243, 1191, 1011. HRMS [M + H]^+^ calcd for C_15_H_17_ClN_3_O_4_, 338.0908, exp 338.0914.
*N-(2-chloro-5-methoxy-4-(1-methyl-5-oxo-4,5-dihydro-1H-pyrazol-3-yl)phenyl)acetamide***9b.** A total of 250 mg of compound **11** (0.742 mmol) and 21 mg of LiOH (0.890 mmol, 1.2 eq) were dissolved in 14 mL of EtOH/water (5/2). The reactional mixture was stirred at room temperature for 1 h 45 min. After evaporation in vacuo, water was added, adjusted at pH 4–5 and extracted 3 times with CHCl_3_. The organic phases were combined, washed once with water, dried on MgSO_4_, and concentrated to obtain 176 mg of monoprotected compound 9b and used without further purification. Yield = 76%; m.p. = 219 -221 °C; ^1^H-NMR (CDCl_3_, 400 MHz): 8.26 (s, 1H), 7.94 (s, 1H), 7.73 (br s, 1H), 3.88 (s, 3H), 3.74 (s, 2H), 3.39 (s, 3H), 2.27 (s, 3H); ^13^C-NMR (CDCl_3_, 100 MHz) 172.6, 168.6, 156.8, 151.6, 136.9, 126.9, 116.2, 114.0, 104.1, 56.0, 42.0, 31.3, 25.2; MS [M + H]^+^ = 295.85; IR (KBr, cm^−1^): 3316, 2340, 1681, 1606, 1586, 1547, 1506, 1450, 1392, 1209, 1015. HRMS [M + H]^+^ calcd for C_13_H_15_ClN_3_O_3_, 296.0802, exp 296.0803.

##### General Procedure for the O-Alkylation

The pyrazolone was dissolved in DMF (3 mL·mmol^−1^) and K_2_CO_3_ (2 eq) and *N*-Boc-4-iodomethylpiperidine (1.2 eq) were added. The mixture was stirred for 3 h at 60 °C. After cooling, the brown solution was diluted in water and extracted 3 times with EtOAc. The organic phases were combined, washed 5 times with water, dried on MgSO_4_, and concentrated under reduced pressure. The crude product was then purified on silica gel (eluant DCM to DCM/EtOAc 8/2).

*Tert-butyl 4-(((3-(4-acetamido-5-chloro-2-methoxyphenyl)-1-methyl-1H-pyrazol-5-yl)oxy)methyl)piperidine-1-carboxylate***10b**. From 167 mg of compound **9b** (0.379 mmol), 60 mg of compound **10b** were obtained as a white solid. Yield = 22%; m.p. = 182–184 °C; ^1^H-NMR (CDCl_3_, 400 MHz): 8.19 (s, 1H), 7.89 (s, 1H), 7.69 (br s, 1H), 6.00 (s, 1H), 4.15 (br s, 2H), 3.92 (d, *J* = 6.3 Hz, 2H), 3.89 (s, 3H), 3.66 (s, 3H), 2.73 (t, *J* = 13.0 Hz, 2H), 2.23 (s, 3H), 1.98 (m, 1H), 1.78 (d, *J* = 12.6 Hz, 2H), 1.45 (s, 9H), 1.27 (qd, *J* = 12.1 Hz, *J’* = 4.2 Hz, 2H); ^13^C-NMR (CDCl_3_, 100 MHz): 168.4, 155.7, 154.8, 154.4, 144.7, 134.4, 127.4, 119.3, 113.8, 104.4, 86.0, 79.5, 75.7, 55.9, 43.5 (2C), 36.1, 33.7, 28.6 (2C), 28.5 (3C), 25.1; [M + H]^+^ = 492.76; IR (KBr, cm^−1^): 3420, 2936, 2853, 1690, 1585, 1557, 1528, 1450, 1436, 1365, 1247, 1173, 1147, 1018.
*Tert-butyl 4-(((1-methyl-3-phenyl-1H-pyrazol-5yl)oxy)methyl)piperidine-1-carboxylate***10c.** From 218 mg of compound **9c** (0.647 mmol), 129 mg of compound **10c** were obtained as a yellow solid. Yield = 27%; m.p. = 133–135 °C; ^1^H-NMR (CDCl_3_, 400 MHz): 7.73 (m, 2H), 7.37 (m, 2H), 7.27 (m, 1H), 5.80 (s, 1H), 4.16 (br s, 2H), 3.93 (d, *J* = 6.4 Hz, 2H), 3.68 (s, 3H), 2.75 (t, *J* = 12.8 Hz, 2H), 2.00 (m, 1H), 1.80 (d, *J* = 13.0 Hz, 2H), 1.47 (s, 9H), 1.29 (qd, *J* = 11.8 Hz, *J’* = 4.6 Hz, 2H); ^13^C-NMR (CDCl_3_, 400 MHz): 155.1, 154.8, 149.3, 133.8, 128.5 (2C), 127.6, 125.2 (2C), 82.0, 79.6, 75.9, 43.5 (2C), 36.0, 33.8, 28.6 (2C), 28.5 (3C); MS [M + H]^+^ = 372.01; IR (KBr, cm^−1^): 3426, 2925, 2853, 1742, 1690, 1626, 1558, 1512, 1454, 1422, 1367, 1275.
*Tert-butyl 4-(((3-(3,4-dimethoxyphenyl)-1-methyl-1H-pyrazol-5-yl)oxy)methyl)piperidine-1-carboxylate***10d.** From 151 mg of compound **9d** (0.379 mmol), 80 mg of compound **10d** were obtained as a yellow oil. Yield = 21%; ^1^H-NMR (CDCl_3_, 400 MHz): 7.33 (d, *J* = 2.0 Hz, 1H), 7.22 (d, *J* = 8.2 Hz, *J’* = 2.0 Hz, 1H), 6.86 (d, *J* = 8.3 Hz, 1H), 5.74 (s, 1H), 4.18 (br s, 2H), 3.95 (s, 3H), 3.93 (d, *J* = 6.4 Hz, 2H), 3.90 (s, 3H), 3.67 (s, 3H), 2.75 (t, *J* = 13.0 Hz, 2H), 2.00 (m, 1H), 1.79 (d, *J* = 10.3 Hz, 2H), 1.47 (s, 9H), 1.29 (qd, *J* = 11.7 Hz, *J* = 3.9 Hz, 2H); MS [M + H]^+^ = 431.91;

##### General Procedure for the N-Alkylation

The protected compound (0.167 mmol) was dissolved in DCM (15 mL·mmol^−1^) and TFA (2 mL·mmol^−1^) was added. The solution was stirred for 15 min. After concentration, the residue was dissolved in acetone (for the benzylation) or DMF (for the alkylation) and K_2_CO_3_ (10 eq) and 3-methylbenzyl bromide (1.2 eq) or (bromomethyl)cyclohexane (1.3 eq) were added. The mixture was stirred at room temperature (for the benzylation) or warmed at 110 °C (for the alkylation) for 3 h and then concentrated under reduced pressure. The residue was dissolved in a mixture of NaCl sat/EtOAc. The organic phase was dried and evaporated. The crude was then purified on neutral Al_2_O_3_ (gradient of elution: DCM to DCM/EtOAc 8/2).

*2-chloro-4-(4-((1-(cyclohexylmethyl)piperidin-4-yl)methyl)-1-methyl-1H-pyrazol-3-yl)-5-methoxyaniline***3a.** From 59 mg of compound **2** (0.136 mmol), 18 mg of compound **3a** were obtained as a pale brown gum. Yield = 31%; ^1^H-NMR (CDCl_3_, 400 MHz): 7.17 (s, 1H), 7.15 (s, 1H), 6.34 (s, 1H), 4.09 (br s, 2H), 3.88 (br s, 2H), 3.72 (s, 3H), 2.83 (m, 2H), 2.29 (d, *J* = 6.6 Hz, 2H), 2.10 (m, 2H), 1.78−1.10 (m, 17 H), 0.89−0.80 (m, 2H); ^13^C-NMR (CDCl_3_, 100 MHz): 156.8, 147.4, 143.4, 131.8, 129.4, 119.1, 114.6, 110.7, 98.7, 66.0, 55.6, 54.4 (2C), 38.9, 36.9, 35.1, 32.0 (2C), 31.9 (2C), 31.1, 26.7, 26.1 (2C); MS [M + H]^+^ = 431.02; IR (KBr, cm^−1^): 3438, 2921, 2849, 1622, 1525, 1461, 1445, 1402, 1211. HRMS [M + H]^+^ calcd for C_24_H_36_ClN_4_O, 431.2578, exp 431.2576.
2-chloro-5-methoxy-4-(1-methyl-4-((1-(3-methylbenzyl)piperidin-4-yl)methyl)-1H-pyrazol-3-yl)aniline **3b.** From 41 mg of compound **2** (0.095 mmol), 19 mg of compound **3b** were obtained as a pale yellow gum. Yield = 46%; ^1^H-NMR (CDCl_3_, 400 MHz): 7.18 (t, *J* = 7.6 Hz, 1H), 7.17 (s, 1H), 7.14 (s, 1H), 7.10 (s, 1H), 7.05 (m, 2H), 6.33 (s, 1H), 4.09 (br s, 2H), 3.86 (s, 3H), 3.71 (s, 3H), 3.40 (s, 2H), 2.81 (m, 2H), 2.33 (s, 3H), 2.28 (d, *J* = 6.8 Hz, 2H), 1.82 (td, *J* = 11.8 Hz, *J’* = 2.4 Hz, 2H), 1.55 (d, *J* = 12.7 Hz, 2H), 1.28 (m, 1H), 1.15 (qd, *J* = 12.3 Hz, *J’* = 3.6 Hz, 2H). ^13^C-NMR (CDCl_3_, 100 MHz): 156.8, 147.4, 143.4, 138.4, 137.7, 131.8, 130.0, 129.3, 128.0, 127.6, 126.4, 119.2, 114.6, 110.7, 98.7, 63.6, 55.6, 53.9 (2C), 38.9, 36.9, 32.2 (2C), 31.1, 21.4; MS [M + H]^+^ = 439.74; IR (KBr, cm^−1^): 3434, 2925, 1623, 1525, 1460, 1445, 1402, 1211. HRMS [M + H]^+^ calcd for C_25_H_32_ClN_4_O, 439.2265, exp 439.2268.
N-(2-chloro-4-(4-((1-(cyclohexylmethyl)piperidin-4-yl)methyl)-1-methyl-1H-pyrazol-3-yl)-5-methoxyphenyl)acetamide **6.** From 60 mg of compound **5** (0.126 mmol), 32 mg of compound **6** were obtained as a beige solid. Yield = 46%; m.p. = 148–150 °C; ^1^H-NMR (CDCl_3_, 400 MHz): 8.20 (s, 1H), 7.70 (br s, 1H), 7.31 (s, 1H), 7.16 (s, 1H), 3.87 (s, 3H), 3.80 (s, 3H), 2.77 (d, *J* = 11.8 Hz, 2H), 2.29 (d, *J* = 6.8 Hz, 2H), 2.26 (s, 3H), 2.03 (d, *J* = 7.0 Hz, 2H), 1.74−1.61 (m, 6H), 1.52 (d, *J* = 12.6 Hz, 2H), 1.43 (m, 1H), 1.28−1.08 (m, 7H), 0.87−0.77 (m, 2H); ^13^C-NMR (CDCl_3_, 100 MHz): 168.5, 156.4, 146.8, 135.1, 130.9, 129.6, 119.8, 119.4, 112.9, 103.8, 66.2, 55.8, 54.5 (2C), 38.9, 37.0, 35.3, 32.3 (2C), 32.1 (2C), 31.2, 26.8, 26.2 (2C), 25.2; MS [M + H]^+^ = 472.92; IR (KBr, cm^−1^): 3420, 2921, 2849, 1694, 1625, 1584, 1527, 1449, 1388, 1243; HRMS [M + H]^+^calcd for C_26_H_34_N_3_O_3_, 473.2683, exp 473.2682.
N-(2-chloro-4-(3-(1-(cyclohexylmethyl)piperidin-4-yl)propanoyl)-5-methoxyphenyl)acetamide **7.** From 81 mg of compound **4** (0.185 mmol), 34 mg of compound **7** were obtained as a beige gum. Yield = 42%; ^1^H-NMR (CDCl_3_, 400 MHz): 8.26 (s, 1H), 7.77 (s, 1H), 7.75 (br s, 1H), 3.90 (s, 3H), 2.93 (t, *J* = 7.6 Hz, 2H), 2.83 (d, *J* = 11.0 Hz, 2H), 2.25 (s, 3H), 2.05 (d, *J* = 7.1 Hz, 2H), 1.79–1.45 (m, 10H), 1.23–1.05 (m, 8H), 0.83 (m, 2H); ^13^C-NMR (CDCl_3_, 100 MHz): 200.1, 168.6, 158.4, 138.6, 130.6, 123.5, 113.5, 103.9, 66.2, 55.9, 53.3 (2C), 41.2, 35.7, 35.2, 32.2 (2C), 32.1 (2C), 31.0, 26.8, 26.2 (2C), 25.2; MS [M + H]^+^ = 434.82; IR (KBr, cm^−1^): 3345, 2921, 2849, 1704, 1672, 1599, 1578, 1512, 1450, 1401, 1263, 1241, 1176, 1014; HRMS [M + H]^+^calcd for C_24_H_36_ClN_2_O_3_, 435.2414, exp 435.2415.
N-(2-chloro-5-methoxy-4-(1-methyl-5-((1-(3-methylbenzyl)piperidin-4-yl)methoxy)-1H-pyrazol-3-yl)phenyl)acetamide **12b**. From 70 mg of compound **10b** (0.126 mmol), 45 mg of compound **12b** were obtained as a beige solid. Yield = 64%; m.p. = 174–176 °C; ^1^H-NMR (CDCl_3_, 400 MHz): 8.22 (s, 1H), 7.90 (s, 1H), 7.67 (br s, 1H), 7.21 (t, *J* = 7.4 Hz, 1H), 7.14−7.06 (m, 3H), 6.00 (s, 1H), 3.93 (d, *J* = 6.3 Hz, 2H), 3.91 (s, 3H), 3.67 (s, 3H), 3.48 (s, 2H), 2.94 (d, *J* = 11.2 Hz, 2H), 2.35 (s, 3H), 2.25 (s, 3H), 2.00 (td, *J* = 11.9 Hz, *J’* = 2.5 Hz, 2H), 1.85−1.77 (m 3H), 1.44 (qd, *J* = 11.9 Hz, *J’* = 3.7 Hz, 2H); ^13^C-NMR (CDCl_3_, 100 MHz): 168.4, 155.7, 154.7, 144.7, 138.2, 137.8, 134.4, 130.0, 128.1, 127.8, 127.4, 126.3, 119.4, 113.7, 104.3, 86.0, 76.2, 63.5, 56.0, 53.3 (2C), 35.8, 33.7, 28.9 (2C), 25.1, 21.4; MS [M + H]^+^ = 496.82; IR (KBr, cm^−1^): 3415, 2937, 1694, 1680, 1585, 1557, 1527, 1449, 1361, 1243; HRMS [M + H]^+^calcd for C_27_H_34_ClN_4_O_3_, 497.2319, exp 497.2319.
4-(((1-methyl-3-phenyl-1H-pyrazol-5-yl)oxy)methyl)-1-(3-methylbenzyl)piperidine **12c.** From 62 mg of compound **10c** (0.126 mmol), 20 mg of compound **12c** were obtained as an oil. Yield = 32%; ^1^H-NMR (CDCl_3_, 400 MHz): 7.73 (m, 2H), 7.37 (m, 2H), 7.27 (m, 1H), 7.22 (t, *J* = 7.5 Hz, 1H), 7.15 (s, 1H), 7.13 (d, *J* = 7.5 Hz, 1H), 7.08 (d, *J* = 7.5 Hz, 1H), 5.80 (s, 1H), 3.94 (d, *J* = 6.4 Hz, 2H), 3.68 (s, 3H), 3.52 (s, 2H), 2.98 (d, *J* = 10.7 Hz, 2H), 2.35 (s, 3H), 2.04 (t, *J* = 1.6 HZ, 2H), 1.86 (m, 1H), 1.80 (d, *J* = 13.5 Hz, 2H), 1.48 (m, 2H); ^13^C-NMR (CDCl_3_, 100 MHz): 155.3, 149.3, 137.9, 137.6, 133.9, 130.1, 128.5 (2C), 128.1, 127.9, 127.5, 126.5, 125.2 (2C), 82.0, 76.2, 63.4, 53.2 (2C), 35.7, 33.8, 28.7 (2C), 21.4; MS [M + H]^+^ = 376.00; HRMS [M + H]^+^calcd for C_24_H_30_N_3_O, 376.2389, exp 376.2392.
*4-(((3-(3,4-dimethoxyphenyl)-1_methyl-1H-pyrazol-5-yl)oxy)methyl)-1-(3-methylbenzyl)piperidine***12d**. From 70 mg of compound **10d** (0.126 mmol), 35 mg of compound **12d** were obtained as a pale brown oil. Yield = 67%; ^1^H-NMR (CDCl_3_, 400 MHz): 7.33 (d, *J* = 2.0 Hz, 1H), 7.24−7.19 (m, 2H), 7.14−7.06 (m, 3H), 6.87 (d, *J* = 8.4 Hz, 1H), 5.73 (s, 1H), 3.95 (s, 3H), 3.93 (d, *J* = 6.4 Hz, 2H), 3.90 (s, 3H), 3.67 (s, 3H), 3.49 (s, 2H), 2.95 (d, *J* = 11.7 Hz, 2H), 2.35 (s, 3H), 2.00 (td, *J* = 11.7 Hz, *J’* = 2.4 Hz, 2H), 1.85 (m, 1H), 1.81 (d, *J* = 13.0 Hz, 2H), 1.44 (qd, *J* = 12.3 Hz, *J* = 3.7 Hz, 2H); ^13^C-NMR (CDCl_3_, 100 MHz): 155.3, 149.2, 149.0, 148.7, 138.1, 137.8, 130.0, 128.1, 127.8, 127.1, 126.4, 117.8, 111.0, 108.1, 81.7, 76.3, 63.5, 55.9, 55.9, 53.2 (2C), 35.8, 33.7, 28.8 (2C), 21.4; MS [M + H]^+^ = 435.94; IR (KBr, cm^−1^): 2935, 1609, 1589, 1558, 1525, 1466, 1435, 1258, 1234, 1029; HRMS [M + H]^+^calcd for C_26_H_34_N_3_O_3_, 436.2600, exp 436.2596.

### 3.2. Biological Evaluation

#### 3.2.1. In Vitro Tests of AChE Biological Activity

Inhibitory capacity of compounds on AChE biological activity was evaluated through the use of the spectrometric method of Ellman [24]. Acetylthiocholine iodide and 5,5-dithiobis-(2-nitrobenzoic) acid (DTNB) were purchased from Sigma Aldrich. AChE from human erythrocytes (buffered aqueous solution, ≥500 units/mg protein (BCA), Sigma Aldrich) was diluted in 20 mM HEPES buffer pH 8, 0.1% Triton X-100 such as to have enzyme solution with 0.25 unit/mL enzyme activity. In the procedure, 100 μL of 0.3 mM DTNB dissolved in phosphate buffer pH 7.4 were added into the 96 wells plate followed by 50 μL of test compound solution and 50 μL of enzyme (0.05 U final). After 5 min of preincubation at 25 °C, the reaction was then initiated by the injection of 50 μL of 1 mM acetylthiocholine iodide solution. The hydrolysis of acetylthiocholine was monitored by the formation of yellow 5-thio-2-nitrobenzoate anion as the result of the reaction of DTNB with thiocholine, released by the enzymatic hydrolysis of acetylthiocholine, at a wavelength of 412 nm using a 96-well microplate plate reader (Synergy 2, Biotek, Colmar, France). Test compounds were dissolved in analytical grade DMSO. Donepezil was used as a reference standard. The rate of absorbance increase at 412 nm was followed every minute for 10 min. Assays were performed with a blank containing all components except acetylthiocholine, in order to account for non-enzymatic reaction. The reaction slopes were compared and the percent inhibition due to the presence of test compounds was calculated by the following expression: 100 − (vi/v0 × 100) where vi is the rate calculated in the presence of inhibitor and v0 is the enzyme activity.

First screening of AChE activity was carried out at a 10^−6^ M concentration of compounds under study. For the compounds with significant inhibition (≥50%), IC_50_ values were determined graphically by plotting the % inhibition versus the logarithm of six inhibitor concentrations in the assay solution using the GraphPad Prism 6 software.

#### 3.2.2. Pharmacological Characterization of Drugs on Human 5-HT_4_R

For competition studies, 2.5 µg of proteins (5-HT_4(b)_ membrane preparations, HTS110M, Eurofins. Eurofins’ 5-HT_4(b)_ membrane preparations are crude membrane preparations made from their proprietary stable recombinant cell lines to ensure high-level of GPCR surface expression.) were incubated in duplicate at 25 °C for 60 min in the absence or the presence of 10^−6^ or 10^−8^ M of each drug (RS67333 was used as a reference standard) and 0.5 nM [^3^H]-GR113808 (NET 1152, Perkin Elmer) in 25 mM Tris buffer (pH 7.4, 25 °C). At the end of the incubation, homogenates were filtered through Whatman GF/C filters (Alpha Biotech) presoaked with 0.5% polyethylenimine using a Brandel cell harvester. Filters were subsequently washed three times with 1 mL of ice-cold 25 mM Tris buffer (pH 7.4, 4 °C). Non-specific binding was evaluated in parallel in the presence of 30 µM serotonin.

#### 3.2.3. Pharmacological Characterization of Drugs on Human 5-HT_6_R

Drugs were evaluated through their possibility to compete for the binding of [^3^H]-LSD on membranes of HEK-293 cells transiently expressing the human 5-HT_6_ receptors (ref. RBHS6M, Perkin Elmer). In brief, 4 µg of proteins were incubated at 37 °C for 60 min in duplicate in the absence or the presence of 10^−6^ or 10^−8^ M of each drug (serotonin was used as a reference standard) and 2.5 nM [^3^H]-LSD (ref. NET638250UC, Perkin Elmer), in 25 mM Tris-HCl buffer (pH 7.4) supplemented with 0.5 mM EDTA. At the end of the incubation, the homogenates were then filtered through Whatman GF/C filters and washed five times with ice-cold 25 mM Tris-HCl buffer. Non-specific binding was evaluated in the presence of 100 µM serotonin. Radioactivity associated to proteins was then quantified and expressed as the percentage of inhibition of the drugs under study.

For some of these compounds, affinity constants were calculated from five-point inhibition curves using the GraphPad Prism 6 software and expressed as Ki ± SD.

### 3.3. In Silico Study

The X-ray structure of donecopride was used as a 3D model during the docking and four conformers present in the crystal cell were used. The initial model of compounds **7**, **3b,** and **12b** were built from donecopride X-ray structure [6]. The compound’s protonation state at pH 7.4 was predicted using standard tools of the ChemAxon Package (http://www.chemaxon.com/, accessed on 23 February 2021). The majority microspecie protonated on piperidine nitrogen at this pH was used for docking studies of each compounds.

The crystallographic coordinates of human acetylcholinesterase used in this study were obtained from X-ray structure of the donepezil/AChE complex (PDB ID 4EY7, a structure refined to 2.35 Å with an R factor of 17.7%) [25]. The AChE amino acid protonation state was checked before the docking study using the ProPKA software and the proposed protonation for Glu202 was applied.

The docking of the donepezil, donecopride, and compounds **3b**, **7**, and **12b** into the AChE was carried out with the GOLD program (v2020.2.0) using the default parameters [26,27]. This program applies a genetic algorithm to explore conformational spaces and ligand binding modes. To evaluate the proposed ligand positions, the ChemPLP fitness function was used. The binding site in the AChE model was defined as a 7 Å sphere from the co-crystallized donepezil ligand and a water molecule interacting with protonated piperidine ring of donepezil was conserved during the docking (residue number 931). The second docking with the higher number of docking cycles (20 cycles) was carried out for compound **3b**.

For the docking studies into 5-HT_4_ receptor, the 3D model of the human 5-HT_4_R built previously by homology sequence approach [28] was used. This model was generated using the crystal structure (PBD: 2RH1) of the human β_2_ adrenergic receptor-T4 lysozyme fusion protein complexed with the carazolol [29] as a template. The docking of the donecopride and **7**, **3b**, **12b** compounds into the generated model was carried out with the GOLD program (v2020.2.0) using the default parameters [26,27] and the proposed ligand positions were evaluated by the ChemPLP fitness function. The binding site in the 5-HT_4_R model was defined as a 10 Å sphere centered on the aspartic acid residue Asp_100_. As the mutagenesis studies have shown that the interaction between the positively ionizable amine of ligands and Asp100 of 5-HT_4_R is crucial for ligand binding, a hydrogen bond constraint between positively ionizable amine ligand and OD atom of Asp_100_ was used during the docking [30] Furthermore, special attention was paid during the docking procedure to the following amino acids in the binding site, which were kept flexible: Arg96, Asp100, Thr104, Tyr192, Ser197, and Trp294.

For the docking studies on the 5-HT_6_ receptor first a homology model was built. The sequence of the human 5-HT_6_R was retrieved from the UniProt Knowledgebase (UniProtKB) [31] (ID: P50406_HUMAN). Using screening methods like FUGUE [32], SP3 [33], PSIBLAST, [34,35] HHSEARCH [36], and the @tome-2 server [37], the β_2_ adrenergic receptor was identified as the best 3D experimental template for the homology modeling also for the 5-HT_6_R (Sequence identity = 32%). The high-resolution (2.4 A) crystal structure of the human β_2_ adrenergic receptor (β_2_AR)-T4 lysozyme fusion protein bound to the carazolol (PDB: 2RH1) [29] was used as the 3D template. The alignment between the two sequences was manually optimized to avoid insertions and deletions in secondary structure elements. Disulfide bond: Cys_99_-Cys_180_ between the transmembrane helix 3 (TM3) and the extracellular loop (ECL2) was conserved. This alignment (Figure S2) was used as the basis for the homology modeling with the Modeller software [38]. The resulting model was then evaluated by methods like verify3D [39] and Eval23D [40].

The docking of the donecopride and **7**, **3b**, **12b** compounds into the generated model was carried out with the GOLD program (v2020.2.0) [26,27] using the same procedure as for 5-HT_4_R. The binding site in the 5-HT_6_R model was defined as a 10 Å sphere centered on the cysteine residue Cys_110_. A hydrogen bond constraint between positively ionizable amine ligand and OD atom of Asp_100_ was also applied during the docking and the following amino acids were kept flexible: Trp_102_, Asp_106_, Ser_193_, Thr_196_, and Trp_307_.

## 4. Conclusions

In conclusion, we prepared two novel series of conformationally constrained donecopride analogs using a late stage functionalization process. The introduction of the novel pyrazole ring instead of the ketone linked reduces the ability of novel ligands to interact both with AChE and 5-HT_4_R. These results were confirmed thanks to our docking study. The replacement of the cyclohexyl moiety of donecopride with a *m*-tolyl group led, however, to a novel phenyl pyrazole **3b** as a dual active compound toward AChE and 5-HT_6_R. According to our docking studies, the introduction of a substituted benzyl moiety on the piperidine ring enhanced the ability of **3b** to interact with AChE and 5-HT_6_R active site thanks to stronger hydrophobic interactions. This preliminary result is of clear interest in the journey toward the identification of valuable pleiotropic compounds for the treatment of neurodegenerative diseases. Indeed, the simultaneous modulation of 5-HT_6_R and AChE could lead to an interesting synergistic effect in the treatment of AD, as demonstrated with the use of idalopirdine and donepezil in the clinical trials.

Based on these promising preliminary results, a future pharmacomodulation of **3b** will be envisaged in order to increase its interaction with 5-HT_6_R and with AChE.

## Figures and Tables

**Table 1:** *h*AChE inhibitory activity and affinity for 5-HT_4_R and 5-HT_6_R.

	*h*AChE		*h*5-HT_4_R		*h*5-HT_6_R	
Compound	% Inhibition at 10^−6^ M	IC_50_ (nM) (*n* = 3)	% Inhibition at 10^−6^ M	K*i* (nM) (*n* = 3)	% Inhibition at 10^−6^M	K*i* (nM) (*n* = 3)
Donepezil	98%	7.7 ± 1.3	−	−	−	−
RS67333	−	−	100	5.1 ± 0.51	−	−
Idalopirdine	−	−	−	−	106	7.05 ± 0.61
Donecopride	96%	16 ± 5	100%	7.05 ± 0.61	30%	ND
MR33372	89%	(16 ± 4) × 10	98%	(17 ± 1) × 10	80%	(23 ± 5) × 10
**7**	98%	34 ± 5	94%	(3 ± 1) × 10^2^	−	ND
**3a**	34%	ND	34%	ND	2%	ND
**3b**	76%	110 ± 4	20%	ND	68%	(47 ± 9) × 10
**6**	31%	ND	16%	ND	0%	ND
**12b**	15%	ND	9%	ND	0%	ND
**12c**	15%	ND	10%	ND	6%	ND
**12d**	6%	ND	9%	ND	9%	ND

IC_50_ and K*i* values are expressed as mean ± standard error of the mean (SEM) of three experiments; ND: not determined.

## Data Availability

The data presented in this study are available within the article and Supplementary Materials.

## Supplementary Materials

The following data are available online. Including analytical spectrum of the compounds, superimposition of **7**, **12b** and **3b** with donecopride in AChE binding site and amino-acid sequences alignment of 5-HT_6_R and human β2-adrenergic receptor. Reference [41] is cited in the Supplementary Materials.

## Sample Availability

Samples of the compounds are available from the authors.
