# Supplementary material for: Development of Novel Potential Pleiotropic Compounds of Interest in Alzheimer’s Disease Treatment through Rigidification Strategy
Source: Molecules. 2021 Apr 26;26(9):2536. doi: 10.3390/molecules26092536 (PMC8123621; doi:10.3390/molecules26092536)
Supplement: Supplementary file 1 [file molecules-26-02536-s001.zip › molecules-1143439-supplementary.pdf]

Development of Novel Potential Pleiotropic Compounds of Interest in Alzheimer's Disease Treatment Through Rigidification Strategy.

Cédric Lecoutey [1], Rémi Legay [1], Audrey Davis [1], Jana Sopková-de Oliveira Santos [1], Patrick Dallemagne [1,*] and Christophe Rochais [1,*]

[1] Normandie Univ., Unicaen, Cermn, 14000 CAEN, FRANCE; cedric.lecoutey@unicaen.fr (C.L.), remi.legay@unicaen.fr (R.L.), audrey.davis@unicaen.fr (A.D.), jana.sopkova@unicaen.fr (J.S.), patrick.dallemagne@unicaen.fr (P.D.), christophe.rochais@unicaen.fr (C.R.).

* Correspondence: patrick.dallemagne@unicaen.fr (P.D.); christophe.rochais@unicaen.fr (C.R.); Tel.: +33-2-31-56-68-13 (P.D. and C.R.)

Contents of Supplementary Information

Analytical spectrum of the novel compounds

Superimposition of 7, 12b and 3b with donecopride in AChE binding site

Amino-acid sequences alignment of 5-HT$_6$R and human β2-adrenergic receptor (2RH1)

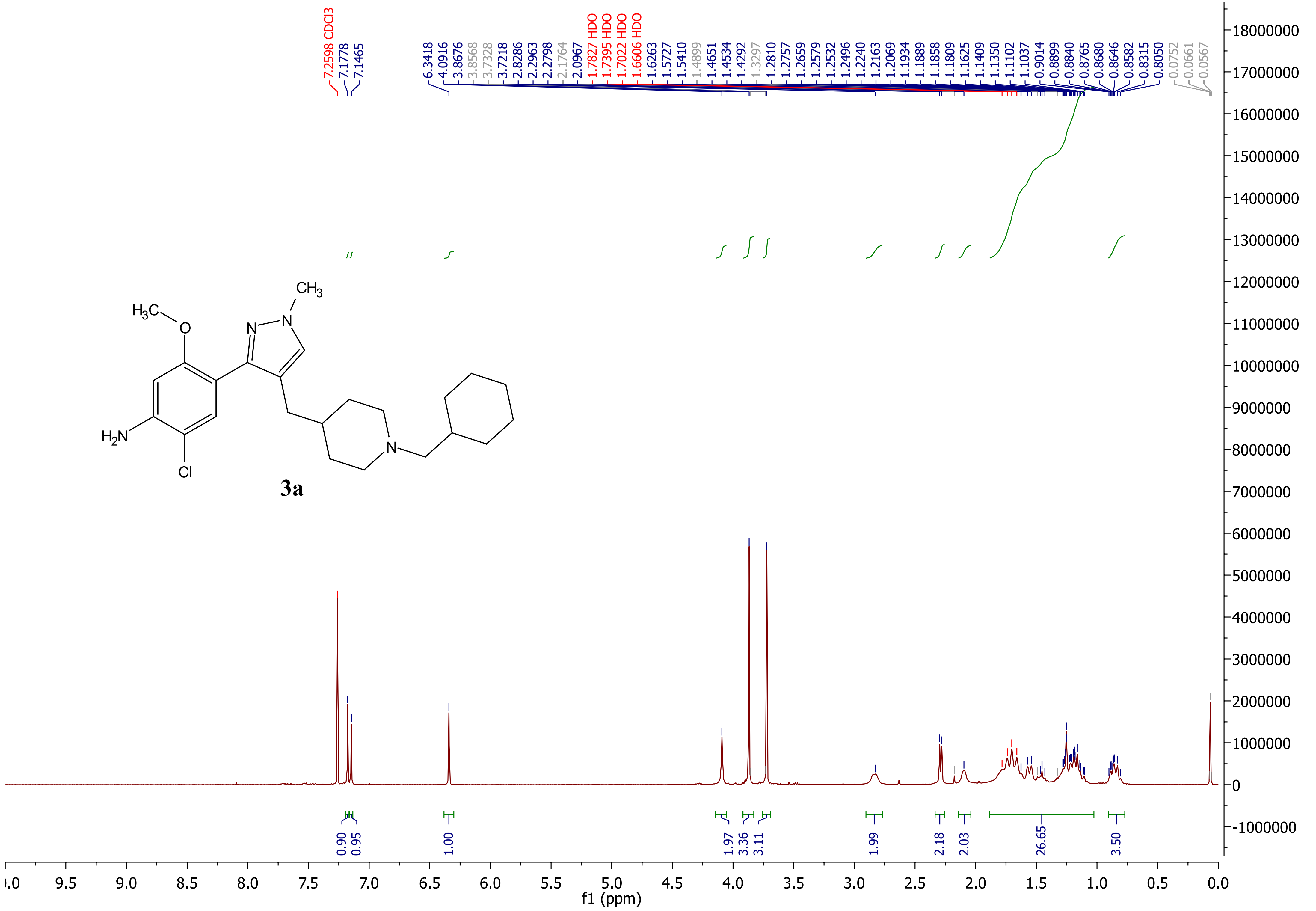
7.2598 CDCl3
7.1778
7.1465
6.3418
4.0916
3.8676
3.8568
3.7328
3.7218
2.8286
2.2963
2.2798
2.1764
2.0967
1.7827 HDO
1.7395 HDO
1.7022 HDO
1.6606 HDO
1.6263
1.5727
1.5410
1.4899
1.4651
1.4534
1.4292
1.3297
1.2810
1.2757
1.2659
1.2579
1.2532
1.2496
1.2240
1.2163
1.2069
1.1934
1.1889
1.1858
1.1809
1.1625
1.1409
1.1350
1.1102
1.1037
0.9014
0.8899
0.8840
0.8765
0.8680
0.8646
0.8582
0.8315
0.8050
0.0752
0.0661
0.0567
H3C
O
N
N
CH3
H2N
Cl
N
3a
0.90
0.95
1.00
1.97
3.36
3.11
1.99
2.18
2.03
26.65
3.50
f1 (ppm)

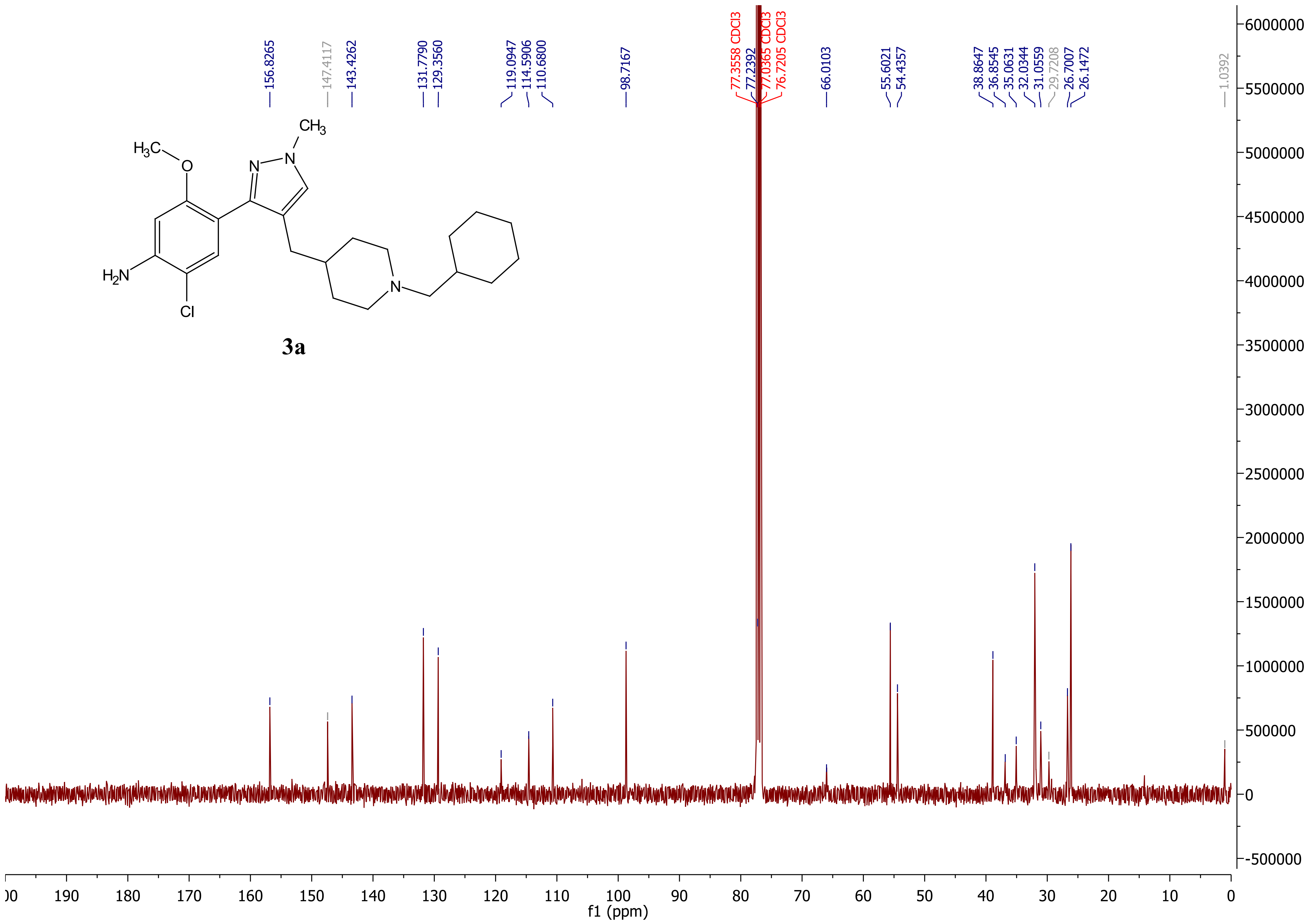
156.8265
147.4117
143.4262
131.7790
129.3560
119.0947
114.5906
110.6800
98.7167
77.3558 CDCl3
77.2392
77.0365 CDCl3
76.7205 CDCl3
66.0103
55.6021
54.4357
38.8647
36.8545
35.0631
32.0344
31.0559
29.7208
26.7007
26.1472
1.0392
3a
f1 (ppm)

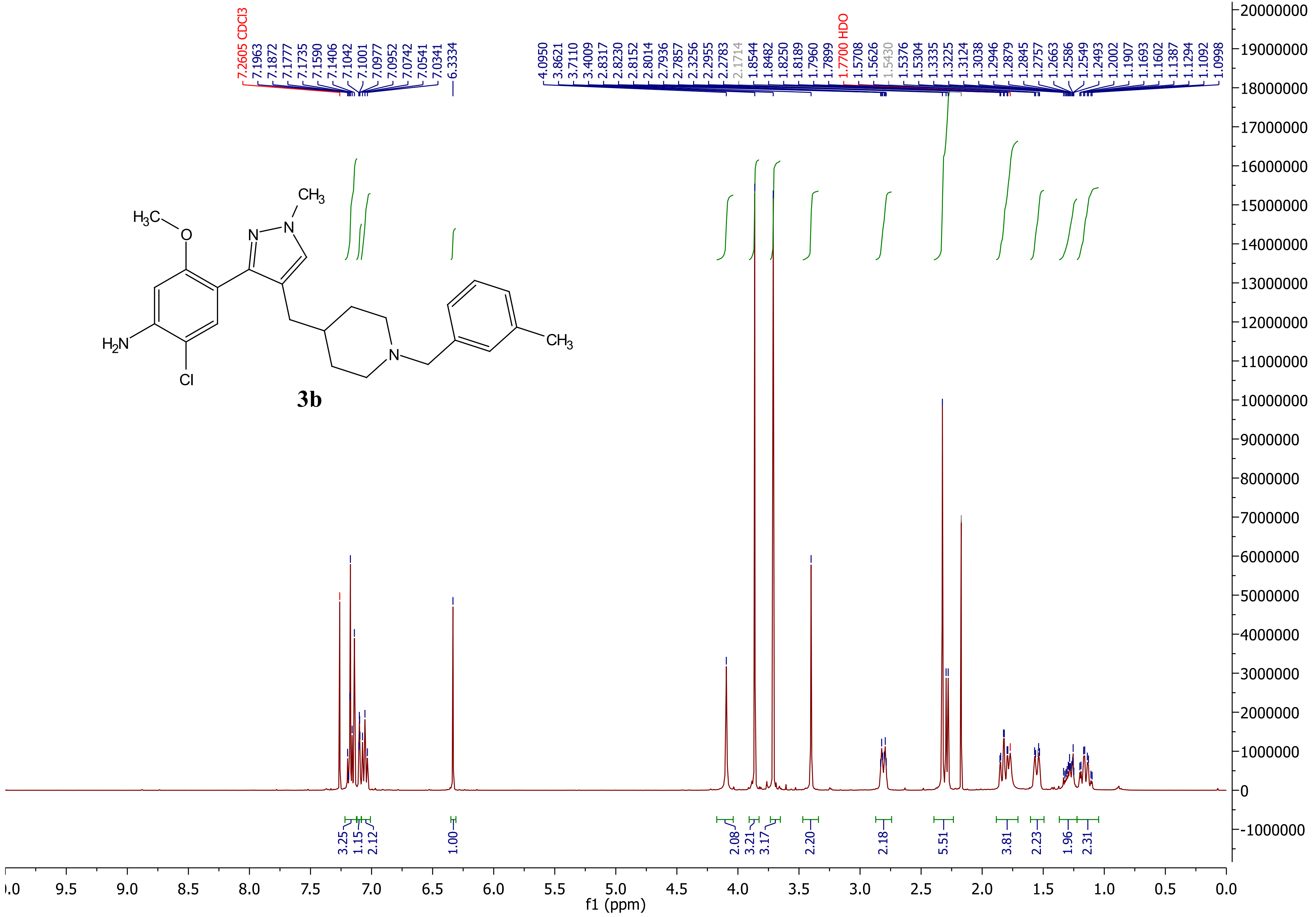

7.2605 CDCl3
7.1963
7.1872
7.1777
7.1735
7.1590
7.1406
7.1042
7.1001
7.0977
7.0952
7.0742
7.0541
7.0341
6.3334
4.0950
3.8621
3.7110
3.4009
2.8317
2.8230
2.8152
2.8014
2.7936
2.7857
2.3256
2.2955
2.2783
2.1714
1.8544
1.8482
1.8250
1.8189
1.7960
1.7899
1.7700 HDO
1.5708
1.5626
1.5430
1.5376
1.5304
1.3335
1.3225
1.3124
1.3038
1.2946
1.2879
1.2845
1.2757
1.2663
1.2586
1.2549
1.2493
1.2002
1.1907
1.1693
1.1602
1.1387
1.1294
1.1092
1.0998
H3C
O
N
N
CH3
H2N
Cl
N
CH3
3b
3.25
1.15
2.12
1.00
2.08
3.21
3.17
2.20
2.18
5.51
3.81
2.23
1.96
2.31
f1 (ppm)

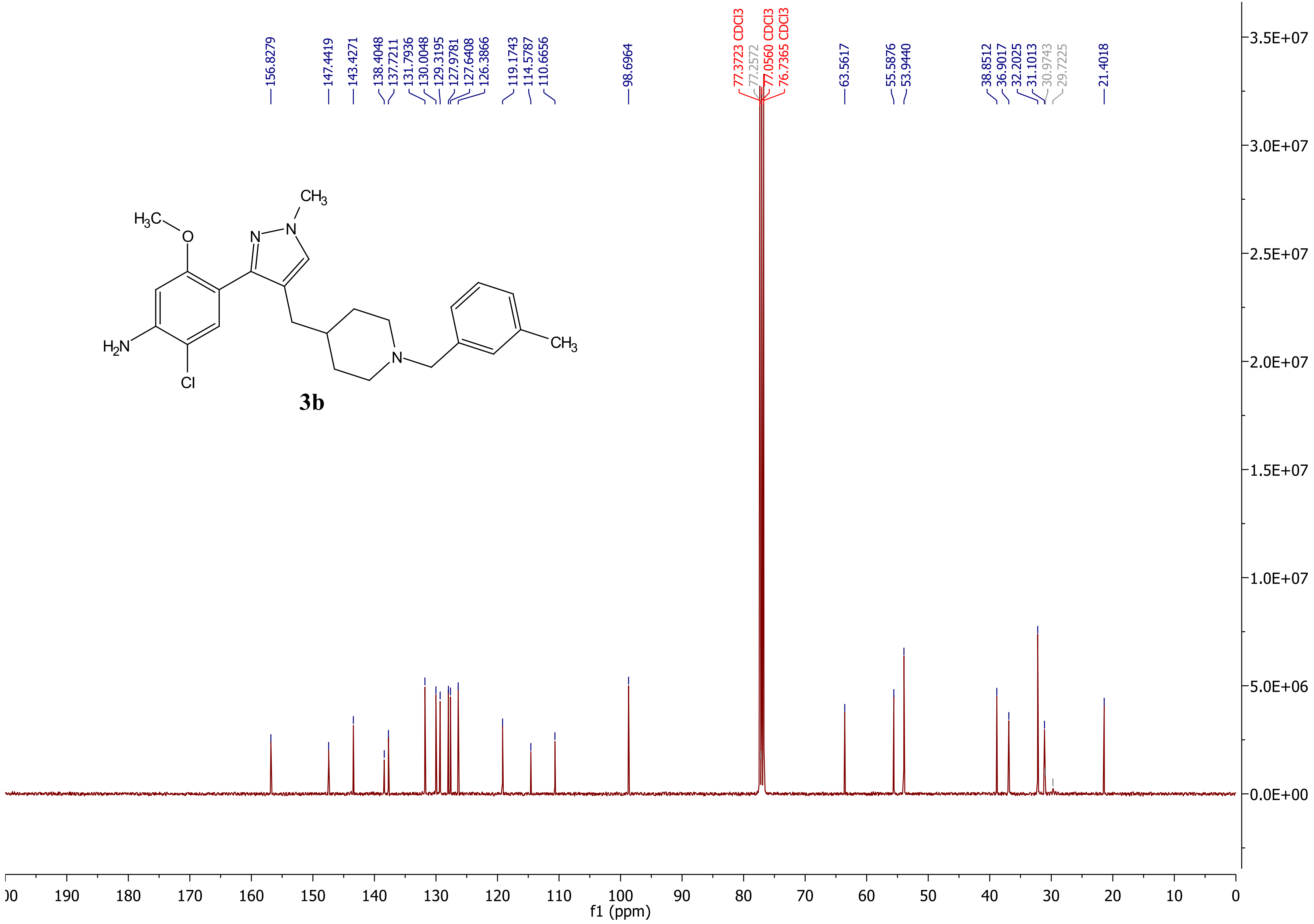
156.8279
147.4419
143.4271
138.4048
137.7211
131.7936
130.0048
129.3195
127.9781
127.6408
126.3866
119.1743
114.5787
110.6656
98.6964
77.3723 CDCl3
77.2572
77.0560 CDCl3
76.7365 CDCl3
63.5617
55.5876
53.9440
38.8512
36.9017
32.2025
31.1013
30.9743
29.7225
21.4018
3.5E+07
3.0E+07
2.5E+07
2.0E+07
1.5E+07
1.0E+07
5.0E+06
0.0E+00
f1 (ppm)

3b

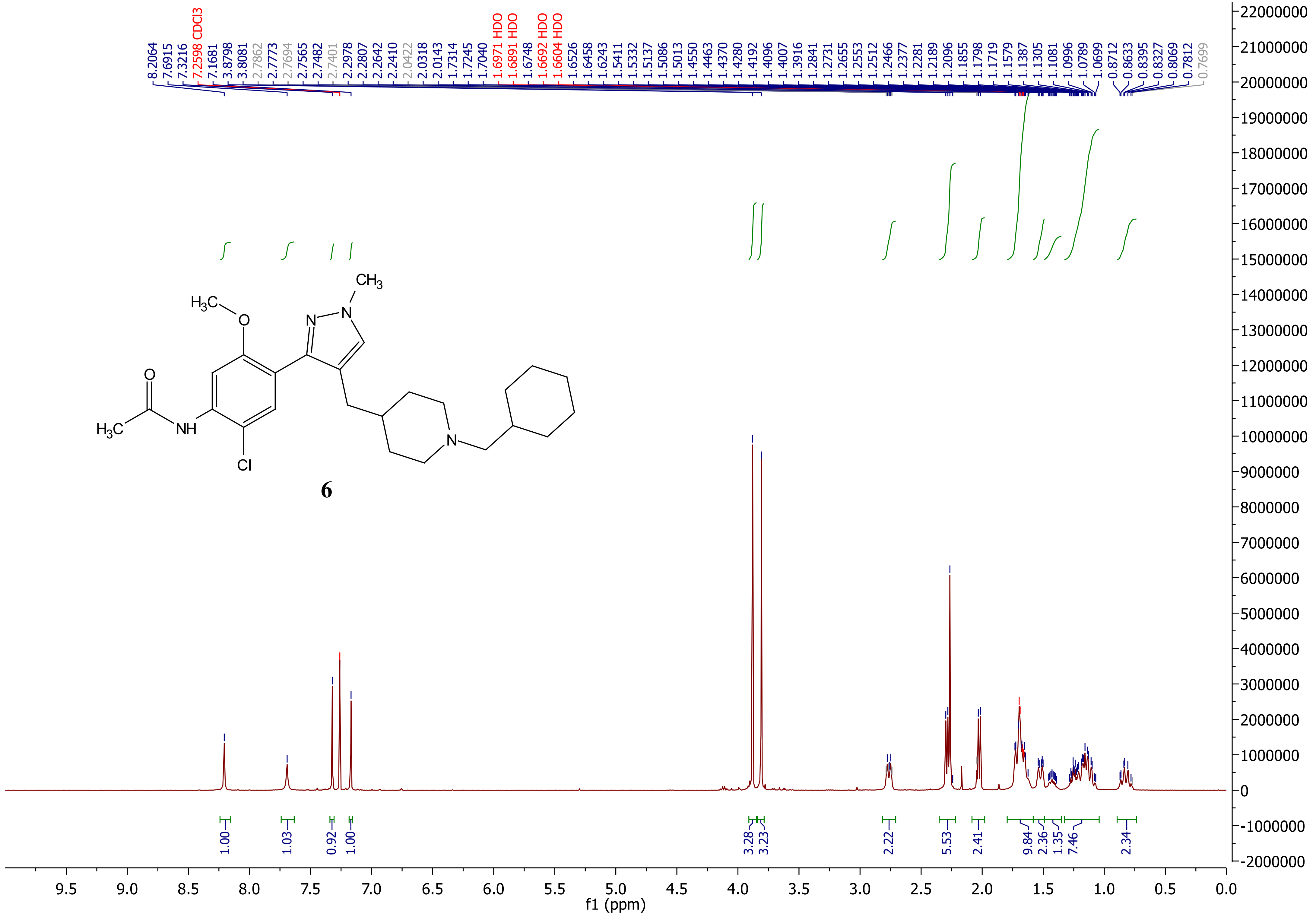
8.2064
7.6915
7.3216
7.2598 CDCl3
7.1681
3.8798
3.8081
2.7862
2.7773
2.7694
2.7565
2.7482
2.7401
2.2978
2.2807
2.2642
2.2410
2.0422
2.0318
2.0143
1.7314
1.7245
1.7040
1.6971 HDO
1.6891 HDO
1.6748
1.6692 HDO
1.6604 HDO
1.6526
1.6458
1.6243
1.5411
1.5332
1.5137
1.5086
1.5013
1.4550
1.4463
1.4370
1.4280
1.4192
1.4096
1.4007
1.3916
1.2841
1.2731
1.2655
1.2553
1.2512
1.2466
1.2377
1.2281
1.2189
1.2096
1.1855
1.1798
1.1719
1.1579
1.1387
1.1305
1.1081
1.0996
1.0789
1.0699
0.8712
0.8633
0.8395
0.8327
0.8069
0.7812
0.7699
H_3C
O
N
N
CH_3
O
H_3C
NH
Cl
N
6
1.00
1.03
0.92
1.00
3.28
3.23
2.22
5.53
2.41
9.84
2.36
1.35
7.46
2.34
f1 (ppm)

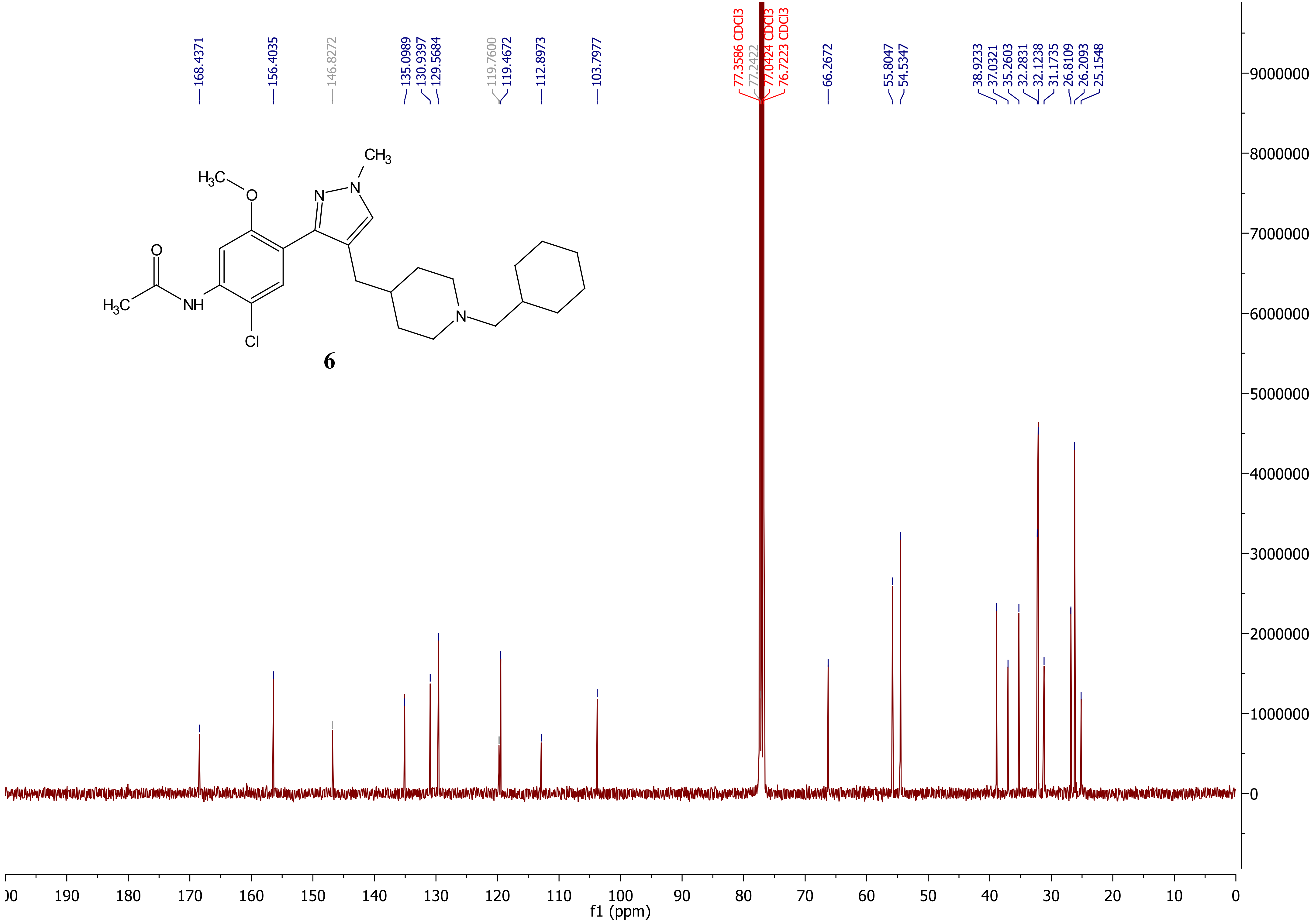

168.4371
156.4035
146.8272
135.0989
130.9397
129.5684
119.7600
119.4672
112.8973
103.7977
77.3586 CDCl3
77.2422
77.0424 CDCl3
76.7223 CDCl3
66.2672
55.8047
54.5347
38.9233
37.0321
35.2603
32.2831
32.1238
31.1735
26.8109
26.2093
25.1548
H_3C
O
NH
Cl
CH_3
N
6
f1 (ppm)

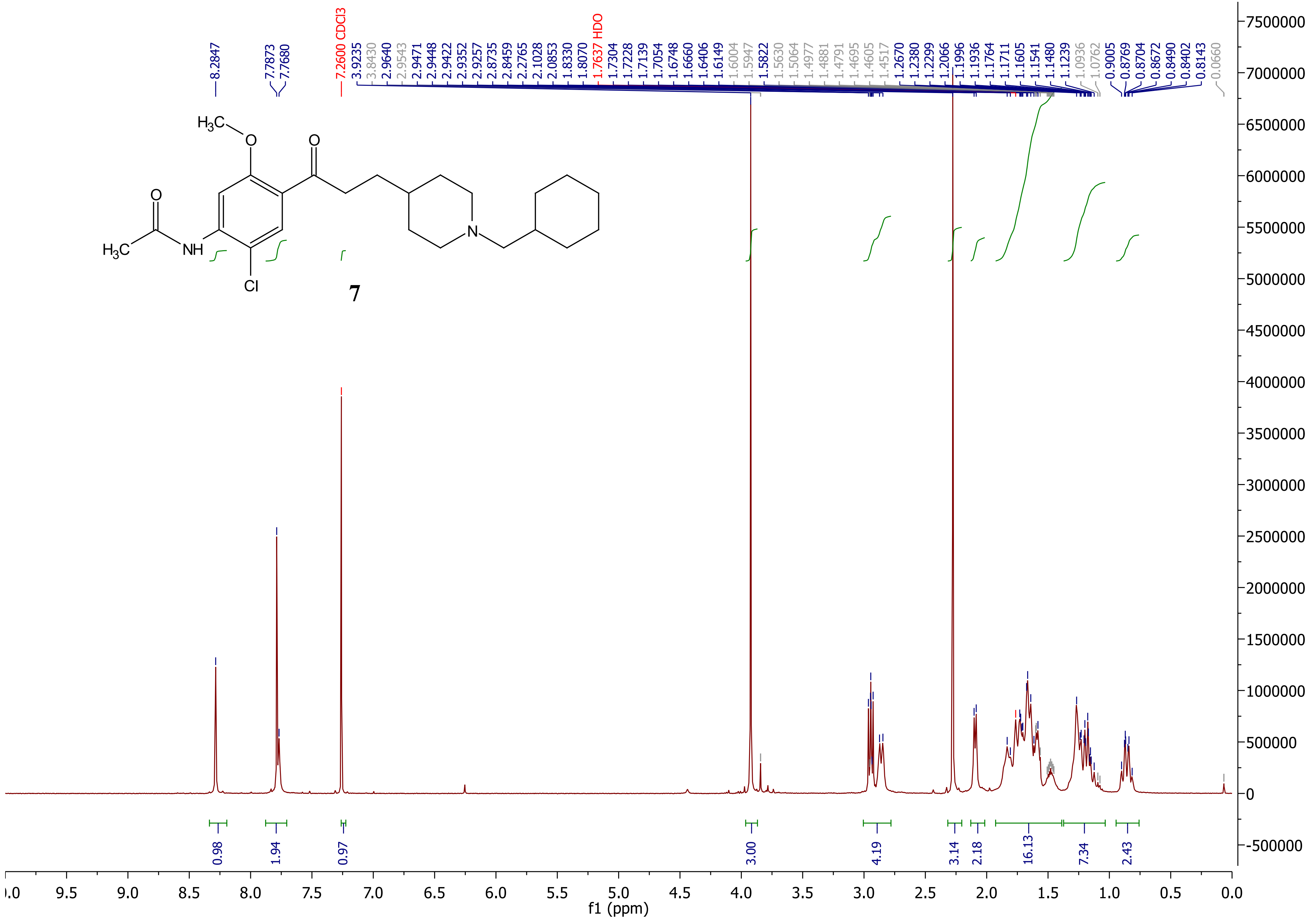
8.2847
7.7873
7.7680
7.2600 CDCl3
3.9235
3.8430
2.9640
2.9543
2.9471
2.9448
2.9422
2.9352
2.9257
2.8735
2.8459
2.2765
2.1028
2.0853
1.8330
1.8070
1.7637 HDO
1.7304
1.7228
1.7139
1.7054
1.6748
1.6660
1.6406
1.6149
1.6004
1.5947
1.5822
1.5630
1.5064
1.4977
1.4881
1.4791
1.4695
1.4605
1.4517
1.2670
1.2380
1.2299
1.2066
1.1996
1.1936
1.1764
1.1711
1.1605
1.1541
1.1480
1.1239
1.0936
1.0762
0.9005
0.8769
0.8704
0.8672
0.8490
0.8402
0.8143
0.0660
H_3C
O
NH
Cl
N
7
0.98
1.94
0.97
3.00
4.19
3.14
2.18
16.13
7.34
2.43
f1 (ppm)

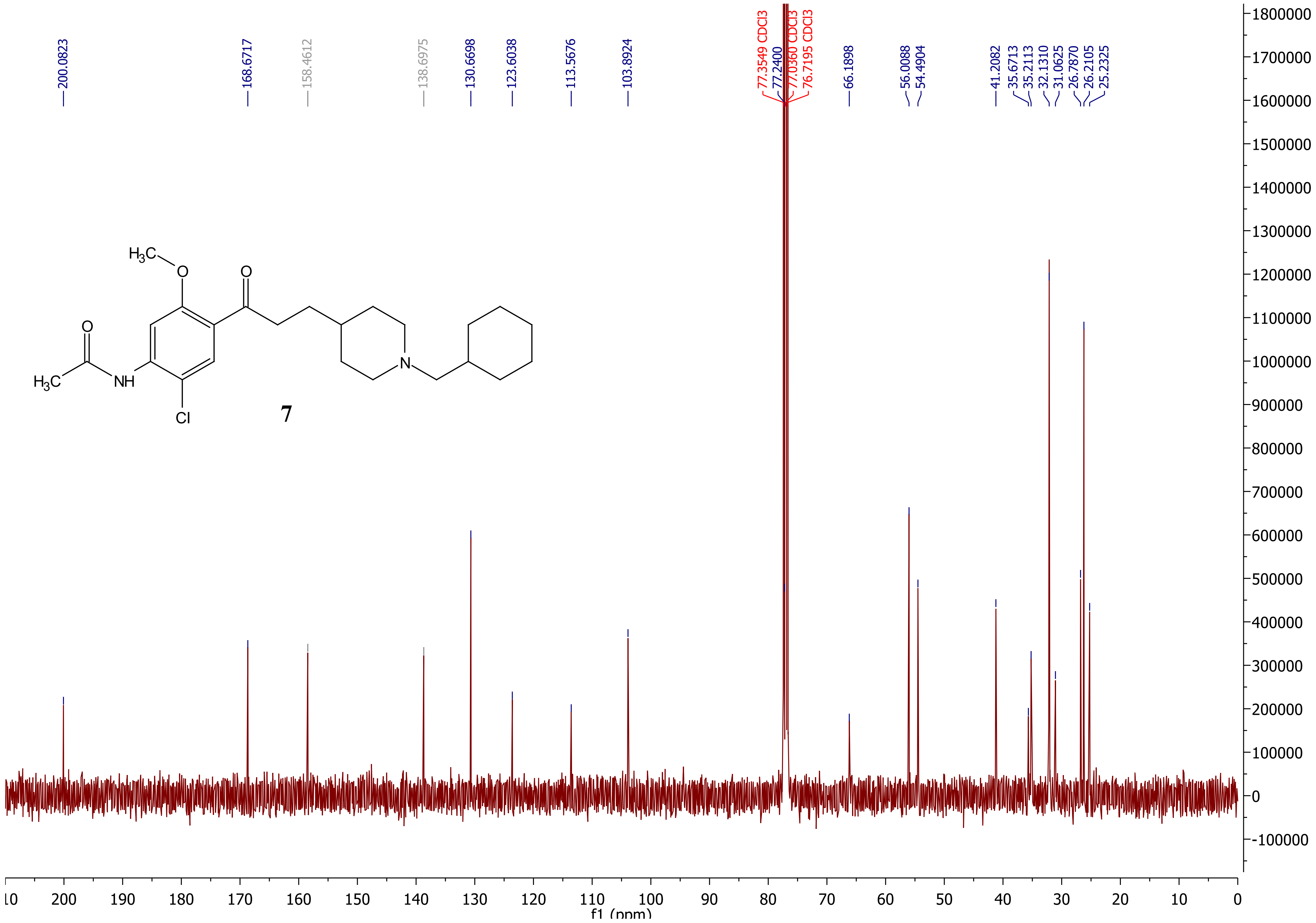
200.0823
168.6717
158.4612
138.6975
130.6698
123.6038
113.5676
103.8924
77.3549 CDCl3
77.2400
77.0360 CDCl3
76.7195 CDCl3
66.1898
56.0088
54.4904
41.2082
35.6713
35.2113
32.1310
31.0625
26.7870
26.2105
25.2325
7
f1 (ppm)

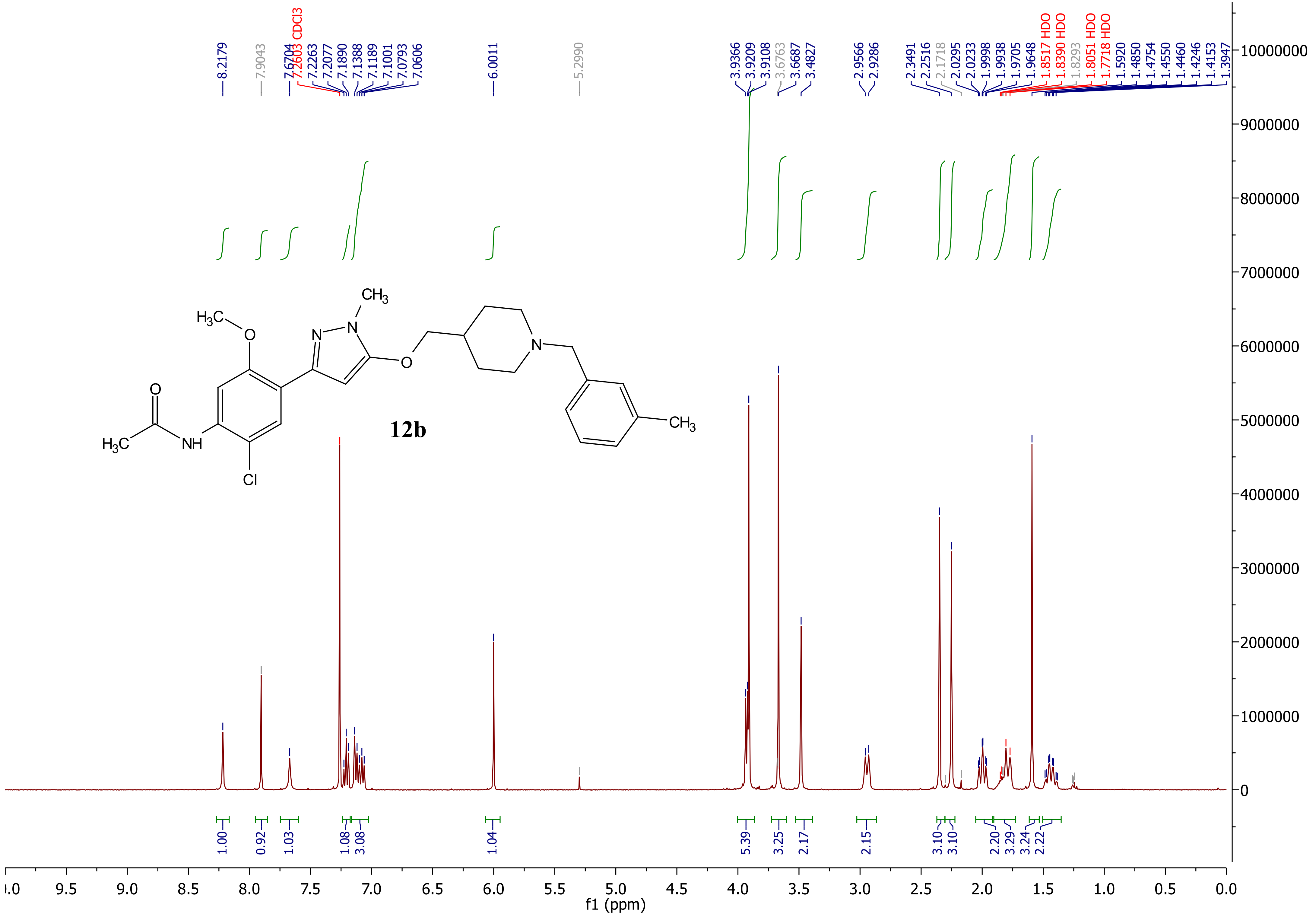

8.2179
7.9043
7.6704
7.2603 CDCl3
7.2263
7.2077
7.1890
7.1388
7.1189
7.1001
7.0793
7.0606
6.0011
5.2990
3.9366
3.9209
3.9108
3.6763
3.6687
3.4827
2.9566
2.9286
2.3491
2.2516
2.1718
2.0295
2.0233
1.9998
1.9938
1.9705
1.9648
1.8517 HDO
1.8390 HDO
1.8293
1.8051 HDO
1.7718 HDO
1.5920
1.4850
1.4754
1.4550
1.4460
1.4246
1.4153
1.3947
1.00
0.92
1.03
1.08
3.08
1.04
5.39
3.25
2.17
2.15
3.10
3.10
2.20
3.29
3.24
2.22
f1 (ppm)

12b

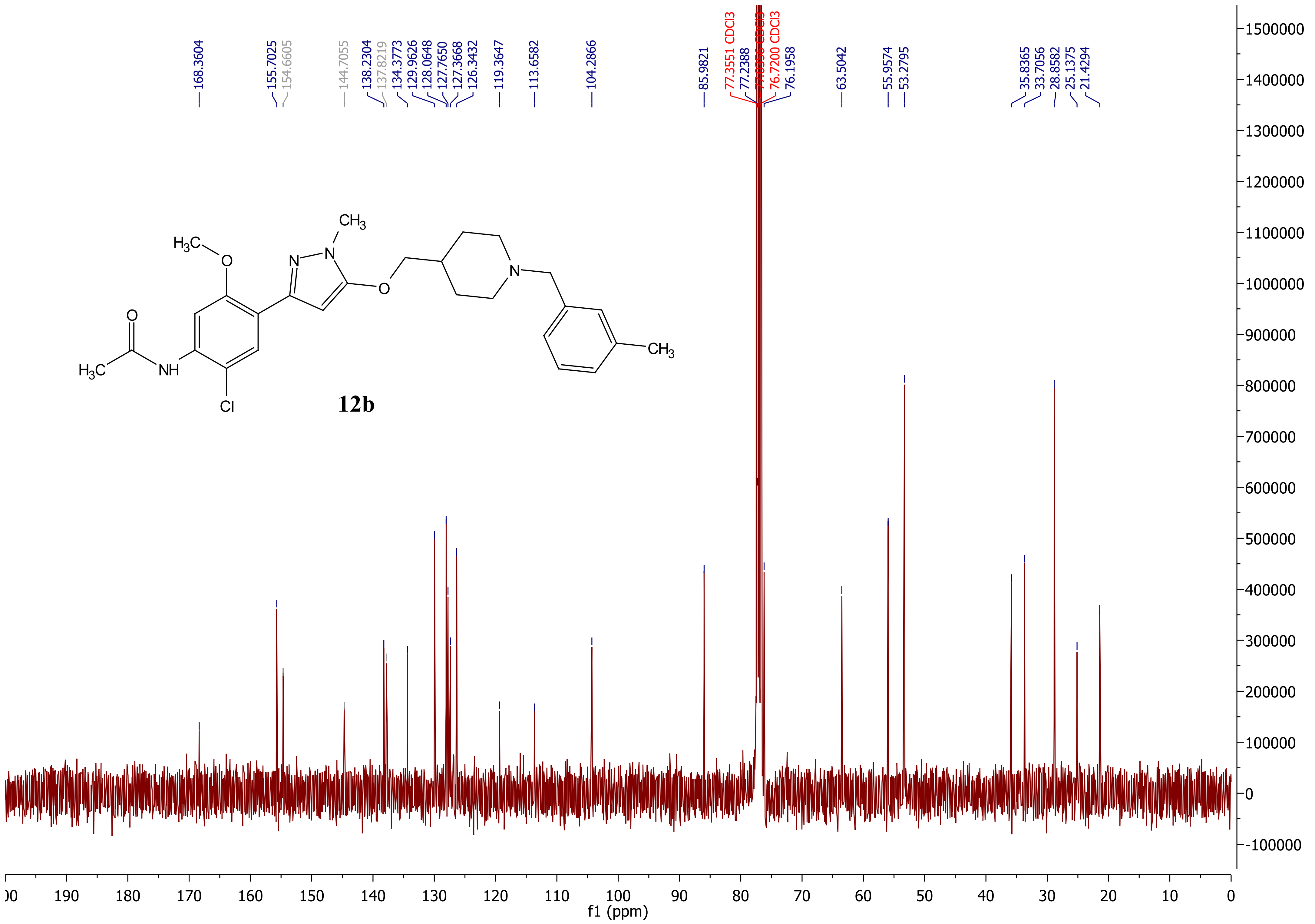

168.3604
155.7025
154.6605
144.7055
138.2304
137.8219
134.3773
129.9626
128.0648
127.7650
127.3668
126.3432
119.3647
113.6582
104.2866
85.9821
77.3551 CDCl3
77.2388
76.7200 CDCl3
76.1958
63.5042
55.9574
53.2795
35.8365
33.7056
28.8582
25.1375
21.4294
12b
f1 (ppm)

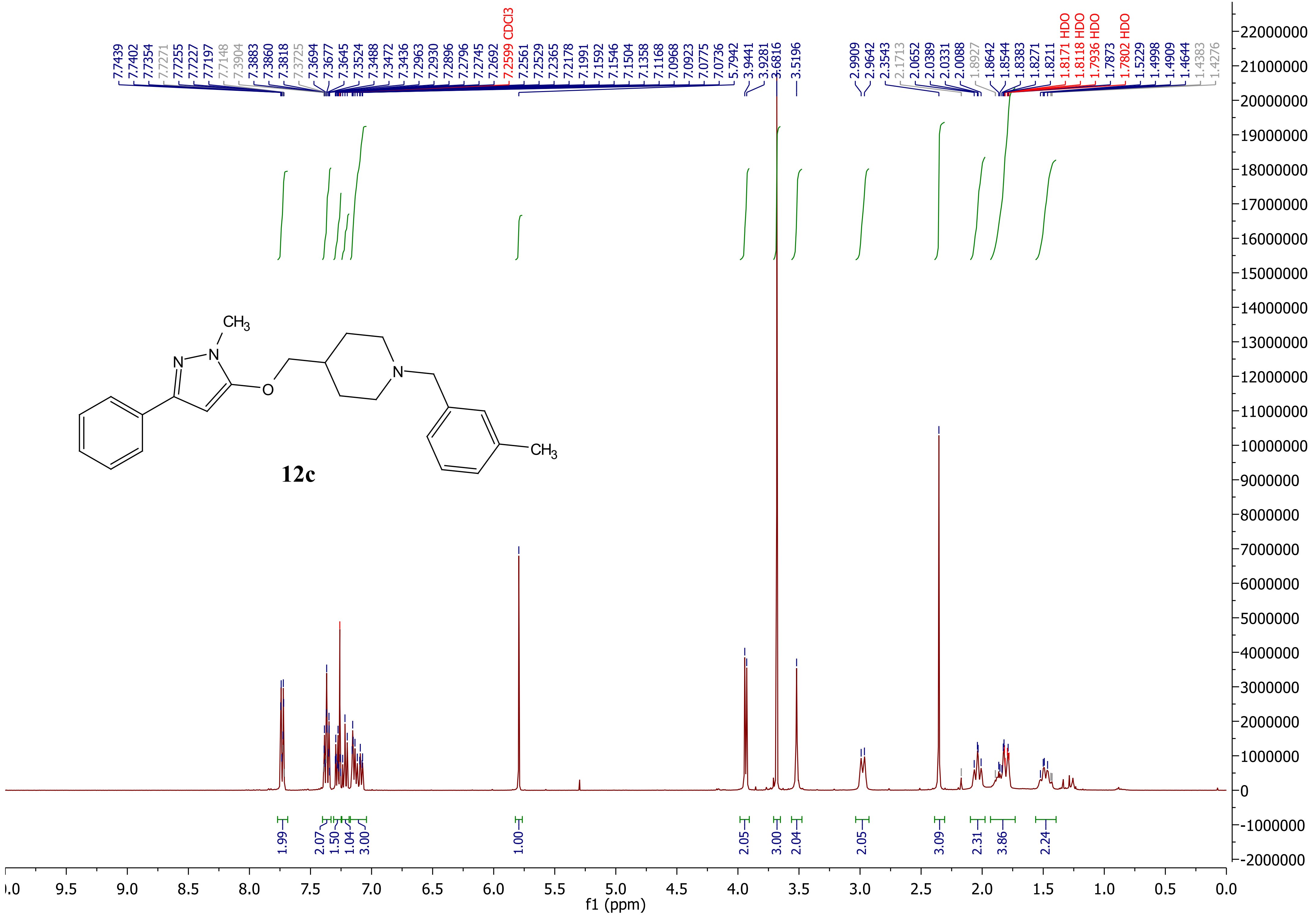
7.7439
7.7402
7.7354
7.7271
7.7255
7.7227
7.7197
7.7148
7.3904
7.3883
7.3860
7.3818
7.3725
7.3694
7.3677
7.3645
7.3524
7.3488
7.3472
7.3436
7.2963
7.2930
7.2896
7.2796
7.2745
7.2692
7.2599 CDCl3
7.2561
7.2529
7.2365
7.2178
7.1991
7.1592
7.1546
7.1504
7.1358
7.1168
7.0968
7.0923
7.0775
7.0736
5.7942
3.9441
3.9281
3.6816
3.5196
2.9909
2.9642
2.3543
2.1713
2.0652
2.0389
2.0331
2.0088
1.8927
1.8642
1.8544
1.8383
1.8271
1.8211
1.8171 HDO
1.8118 HDO
1.7936 HDO
1.7873
1.7802 HDO
1.5229
1.4998
1.4909
1.4644
1.4383
1.4276
CH3
N
N
O
N
CH3
12c
1.99
2.07
1.50
1.04
3.00
1.00
2.05
3.00
2.04
2.05
3.09
2.31
3.86
2.24
f1 (ppm)

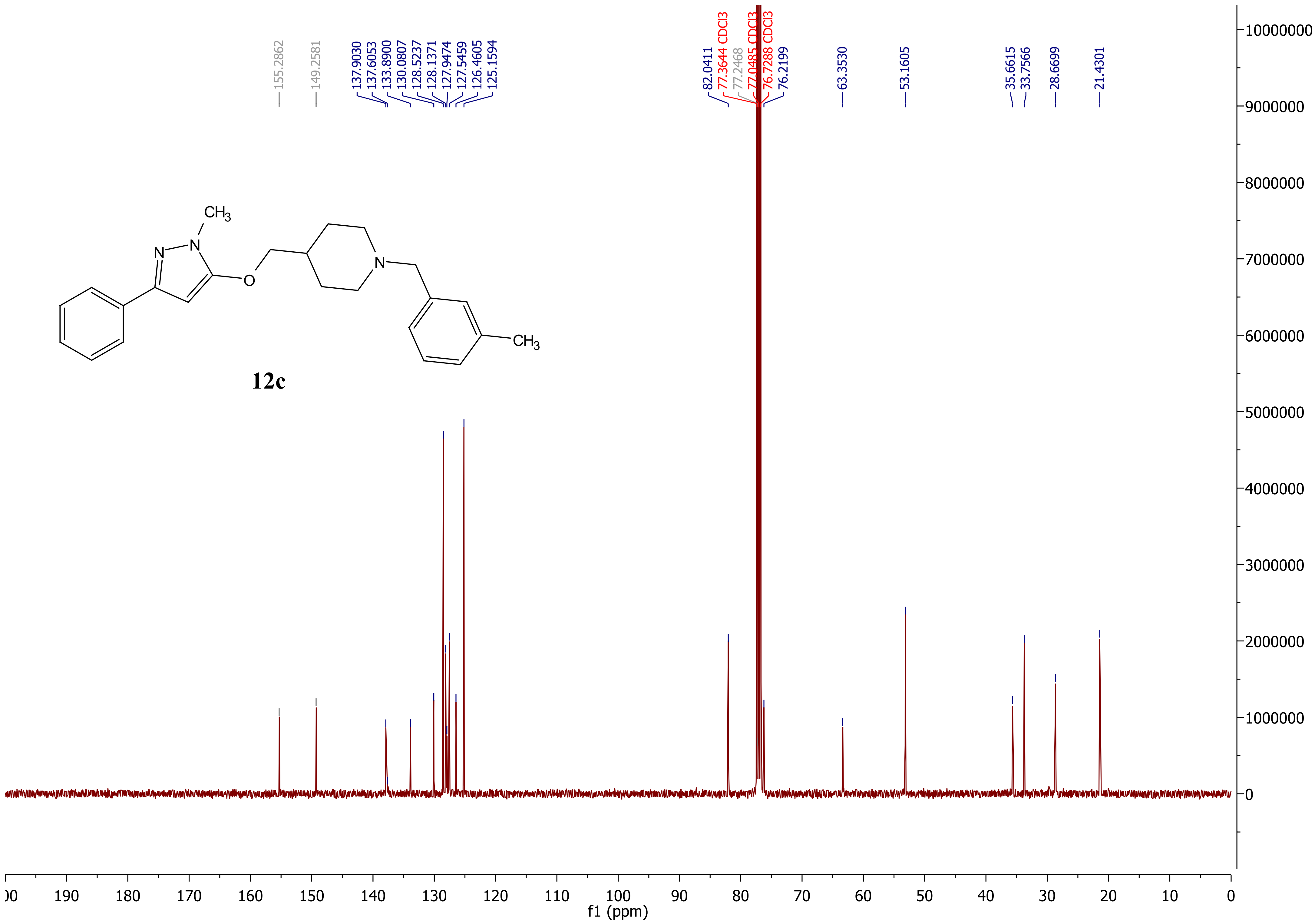
155.2862
149.2581
137.9030
137.6053
133.8900
130.0807
128.5237
128.1371
127.9474
127.5459
126.4605
125.1594
82.0411
77.3644 CDCl3
77.2468
77.0485 CDCl3
76.7288 CDCl3
76.2199
63.3530
53.1605
35.6615
33.7566
28.6699
21.4301
CH3
N
N
O
N
CH3
12c
f1 (ppm)

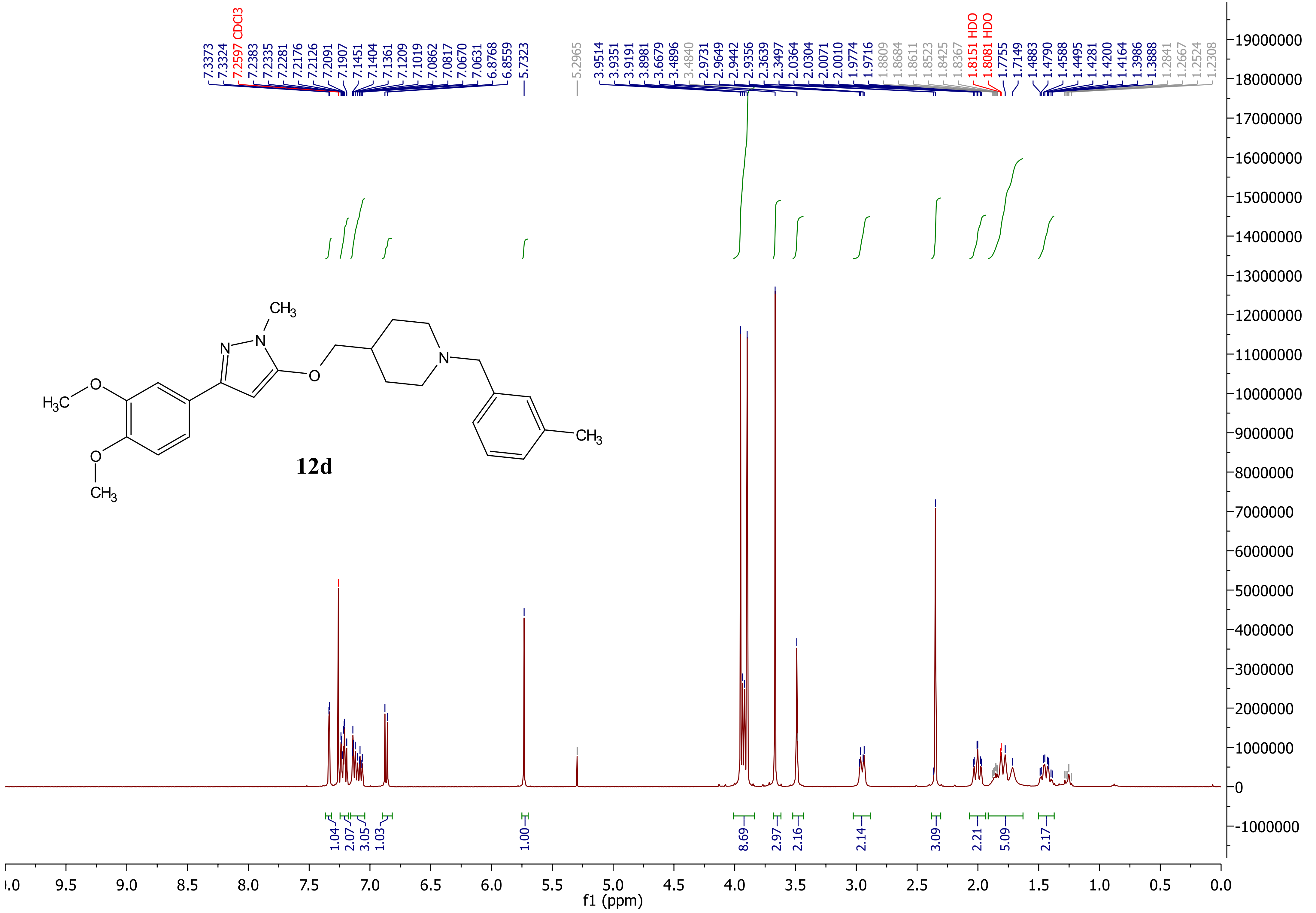
7.3373
7.3324
7.2597 CDCl3
7.2383
7.2335
7.2281
7.2176
7.2126
7.2091
7.1907
7.1451
7.1404
7.1361
7.1209
7.1019
7.0862
7.0817
7.0670
7.0631
6.8768
6.8559
5.7323
5.2965
3.9514
3.9351
3.9191
3.8981
3.6679
3.4896
3.4840
2.9731
2.9649
2.9442
2.9356
2.3639
2.3497
2.0364
2.0304
2.0071
2.0010
1.9774
1.9716
1.8809
1.8684
1.8611
1.8523
1.8425
1.8367
1.8151 HDO
1.8081 HDO
1.7755
1.7149
1.4883
1.4790
1.4588
1.4495
1.4281
1.4200
1.4164
1.3986
1.3888
1.2841
1.2667
1.2524
1.2308
1.04
2.07
3.05
1.03
1.00
8.69
2.97
2.16
2.14
3.09
2.21
5.09
2.17
12d
f1 (ppm)

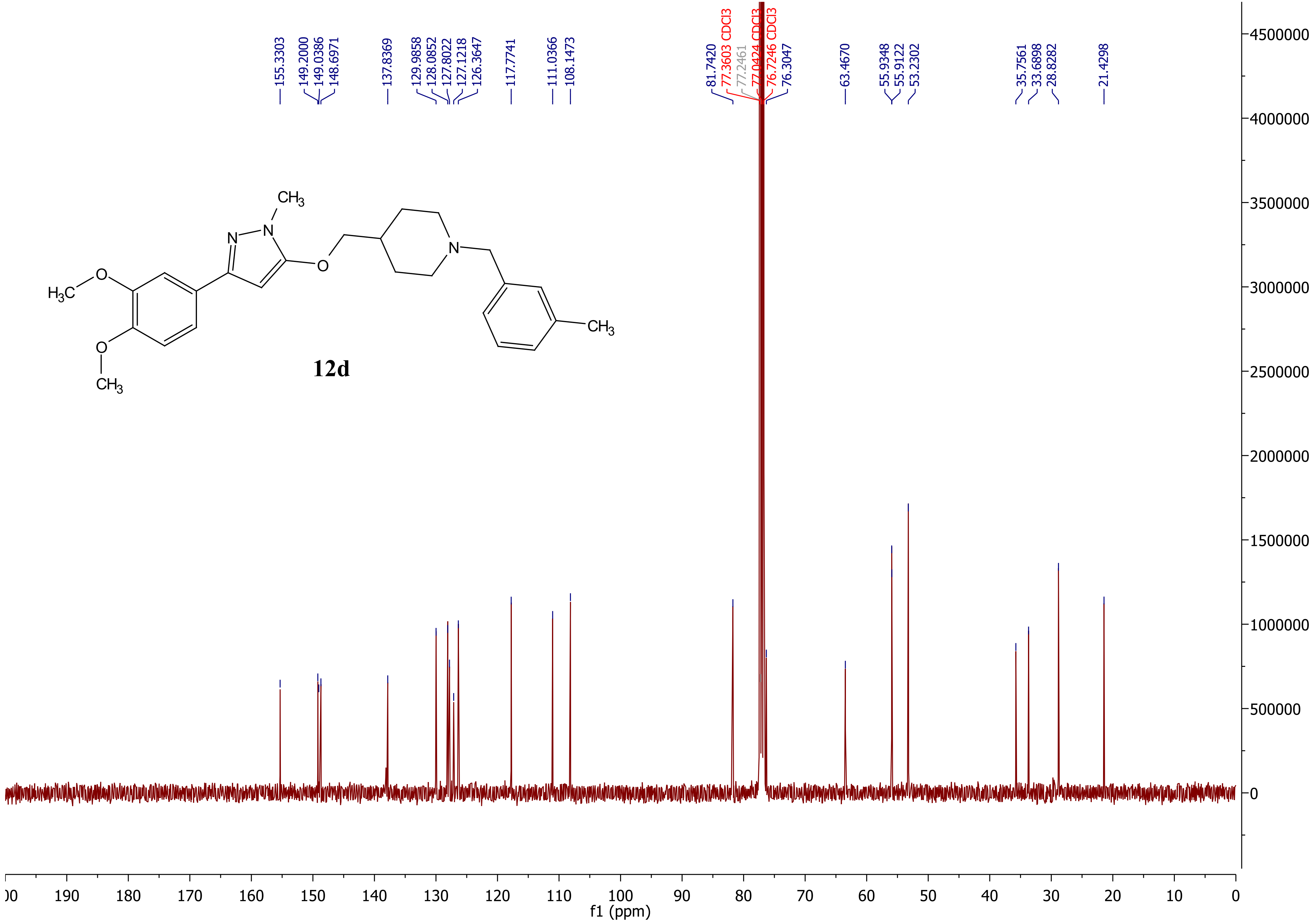
155.3303
149.2000
149.0386
148.6971
137.8369
129.9858
128.0852
127.8022
127.1218
126.3647
117.7741
111.0366
108.1473
81.7420
77.3603 CDCl3
77.2461
77.0424 CDCl3
76.7246 CDCl3
76.3047
63.4670
55.9348
55.9122
53.2302
35.7561
33.6898
28.8282
21.4298
12d
4500000
4000000
3500000
3000000
2500000
2000000
1500000
1000000
500000
0
190
180
170
160
150
140
130
120
110
100
90
80
70
60
50
40
30
20
10
0
f1 (ppm)

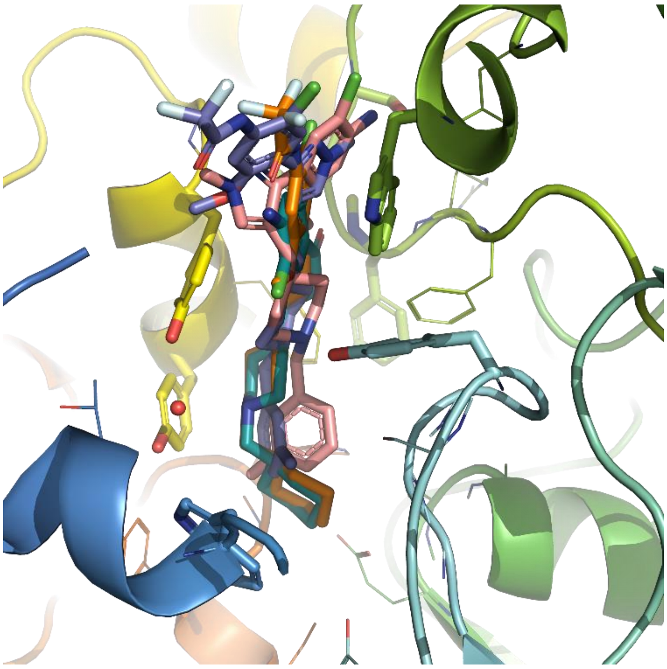

Figure S1. Superposition of **7** (orange), **12b** (purple), **3b** (pink) and Donecopride (yellow) in AChE binding site. This figure was made with PYMOL (DeLano Scientific, 2002, San Carlo, USA)

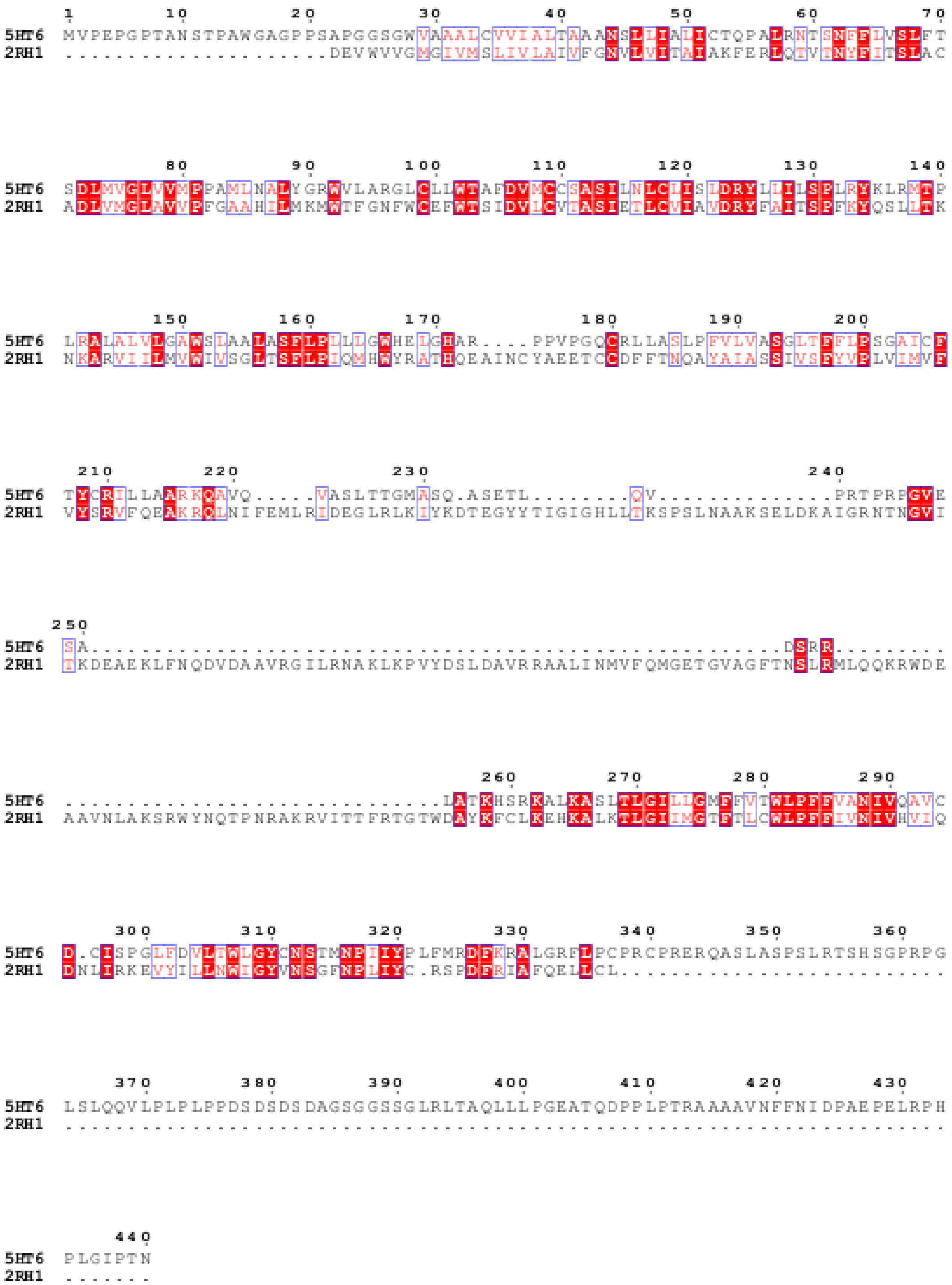

Figure S2. Amino-acid sequences alignment of 5-HT6R and human β2-adrenergic receptor (2RH1). Transmembrane helices (TM1 to TM7) are delimited by orange rectangle. Disulfide bridge between Cys99 and Cys180 is indicated in grey. The figure alignment was made with ESPript (ESPript - https://espript.ibcp.fr) [1].

[1] Robert, X.; Gouet, P. Deciphering key features in protein structures with the new ENDscript server. Nucl. Acids Res. **2014**, *42(W1)*, W320-W324. https://doi:10.1093/nar/gku316
